# Potato purple top phytoplasma infection induces autophagy-associated lipid dynamics that support pathogen proliferation

**DOI:** 10.1093/plphys/kiag628

**Published:** 2026-08-25

**Authors:** Junichi Inaba, Bo Min Kim, Yan Zhao, Qi Huang, Wei Wei

**Affiliations:** Molecular Plant Pathology Laboratory, Beltsville Agricultural Research Center, Agricultural Research Service, United States Department of Agriculture, 10300 Baltimore Ave., Beltsville, MD 20705, United States; Molecular Plant Pathology Laboratory, Beltsville Agricultural Research Center, Agricultural Research Service, United States Department of Agriculture, 10300 Baltimore Ave., Beltsville, MD 20705, United States; Molecular Plant Pathology Laboratory, Beltsville Agricultural Research Center, Agricultural Research Service, United States Department of Agriculture, 10300 Baltimore Ave., Beltsville, MD 20705, United States; Floral and Nursery Plants Research Unit, United States National Arboretum, Agricultural Research Service, United States Department of Agriculture, 10300 Baltimore Ave., Beltsville, MD 20705, United States; Molecular Plant Pathology Laboratory, Beltsville Agricultural Research Center, Agricultural Research Service, United States Department of Agriculture, 10300 Baltimore Ave., Beltsville, MD 20705, United States

## Abstract

Phytoplasmas are unculturable, phloem-restricted bacterial pathogens responsible for devastating diseases in crops and ornamentals worldwide. Their mechanism for nutrient acquisition from host plants remains largely unknown. This study demonstrated that infection with potato purple top phytoplasma induced extensive remodeling of lipid metabolism in tomato plants, closely linked to autophagy activation. Western blot and confocal analyses revealed increased ATG8 lipidation and autophagosome formation at endoplasmic reticulum stress sites, alongside the redistribution of lipid droplets toward phytoplasma cells. Lipidomic profiling showed a decline in chloroplast galactolipids and phospholipids with a concomitant rise in triacylglycerol, indicating accelerated membrane turnover and neutral lipid sequestration. Transmission electron microscopy further revealed frequent spatial proximity between lipid droplets and phytoplasmas. Inhibition of autophagy with 3-methyladenine blocked lipid droplet breakdown, disrupted endoplasmic reticulum organization, and reduced phytoplasma titers, suggesting that host autophagy contributes to phytoplasma proliferation. In addition, genome analysis identified a conserved phytoplasma-encoded alpha/beta hydrolase (potato purple top-lipase), predicted to be related to monoacylglycerol lipases. In vivo assays in yeast and *Nicotiana benthamiana* confirmed that potato purple top-lipase reduced neutral lipids, mainly triacylglycerol, and that catalytic triad mutations abolished activity. Because potato purple top-lipase lacks a predicted secretory signal peptide, it likely functions intracellularly within phytoplasma cells and may participate in the metabolism of lipid intermediates. These findings support a model in which phytoplasma infection is associated with host autophagy-associated lipid droplet mobilization and a phytoplasma lipase that may contribute to host-derived lipid resources, providing insight into potential nutrient acquisition strategies of phloem-restricted pathogens.

## Introduction

Intracellular pathogens have evolved sophisticated strategies to exploit host cellular pathways and metabolic networks, enabling them to survive, proliferate, and persist within challenging host cell environments (Birmingham et al. 2006; Deretic et al. 2013; Escoll et al. 2016). Among these strategies, the manipulation of host autophagy, a conserved eukaryotic degradation and recycling process, has emerged as a common mechanism by which pathogens secure nutrients, modulate immunity, and remodel host organelles to their advantage (Deretic et al. 2013; Huang and Brumell 2014). Autophagy is activated under various stress conditions, including nutrient deprivation, oxidative stress, and endoplasmic reticulum (ER) dysfunction, and involves the formation of double-membrane vesicles known as autophagosomes that engulf cytoplasmic components for degradation and recycling (Mizushima and Komatsu 2011). Lipidation of the ubiquitin-like protein autophagy-related gene 8 (ATG8) and its association with the autophagosomal membrane, together with the conjugation of ATG12 to ATG5 and the formation of the ATG12–ATG5–ATG16L1 complex, are essential steps in autophagosome biogenesis and serve as widely used molecular markers of autophagic activity (Mizushima and Komatsu 2011; Galluzzi et al. 2017).

Phytoplasmas are obligate intracellular bacteria that inhabit plant phloem tissues and are transmitted by insect vectors. By hijacking meristem fate, phytoplasma prioritizes vegetative growth over reproduction, altering plant architecture and resulting in significant agricultural losses worldwide (Hogenhout et al. 2008; Wei et al. 2013). As of 2024, nearly 1,000 plant species are affected by phytoplasmas (Wei et al. 2024). The highly reduced genomes, marked by the loss of key metabolic and biosynthetic functions, reflect long-term adaptation to a host-dependent intracellular lifestyle and render them reliant on host cells for nutrients, growth, and replication (Oshima et al. 2004; Tan et al. 2021). However, because phytoplasmas cannot be cultured in vitro, the mechanisms by which they manipulate host cellular and metabolic processes to acquire nutrients are still not well understood.

Emerging evidence indicates that phytoplasma infection causes widespread reprogramming of host cell functions. Several well-characterized phytoplasma effectors, including TENGU, SAP11, and SAP54/PHYL1, manipulate plant developmental and defense pathways, leading to symptoms such as witches' broom, dwarfism, altered leaf development, and phyllody (Hoshi et al. 2009; MacLean et al. 2011; Sugio et al. 2011; Maejima et al. 2014; Drcelic et al. 2024). In addition to these developmental effects, phytoplasma infection has also been associated with disrupted carbohydrate metabolism, damaged chloroplast degradation, and ER stress (Wei et al. 2022; Inaba et al. 2023; Musetti et al. 2023). Autophagosome-like bodies were often visualized near injured chloroplasts (Wei et al. 2022). The activation of the unfolded protein response (UPR), a conserved signaling pathway that maintains ER health under stress, has been observed during phytoplasma infection (Inaba et al. 2023; Musetti et al. 2023). Autophagy and lipid metabolism are interconnected during plant stress responses. Degradation of damaged organelles, particularly chloroplasts, can generate free fatty acids that are stored as triacylglycerols (TAGs) in lipid droplets (LDs), while ER stress often enhances membrane synthesis and remodeling (Fan et al. 2019; Yoshitake et al. 2019; Münzner et al. 2022). Many intracellular pathogens in both plant and animal systems exploit these processes to create nutrient-rich niches, either by redirecting autophagy-derived metabolites (Steele et al. 2015; Dermastia et al. 2023) or by manipulating host lipid pools (Knight et al. 2018). Whether phytoplasmas employ similar strategies to exploit host autophagy and lipid metabolism remains unexplored.

To address this knowledge gap, this study investigated the interactions among autophagy, lipid remodeling, and pathogen proliferation in tomato plants infected with the potato purple top (PPT) phytoplasma using western blot, cytological, and lipidomic analyses. Our results demonstrated that PPT infection triggered an autophagic response, characterized by ATG8 lipidation and the accumulation of ATG8- and ATG12-positive autophagosomes that partially colocalized with ER stress aggregates. Concurrently, the infection induced extensive remodeling of host lipid metabolism, including chloroplast membrane degradation and the formation of TAG-rich LDs. Furthermore, chemical inhibition of autophagy disrupted LD breakdown and distribution, impaired ER remodeling, and reduced pathogen load. These observations suggest that host autophagy contributes to lipid mobilization during infection. Additionally, a PPT-encoded lipase was identified that, when expressed in yeast and in planta, significantly reduced neutral lipid levels, indicating that the lipase may participate in the metabolism of lipid intermediates. These findings support a model in which phytoplasma infection is associated with host autophagy-mediated lipid remodeling, providing new insight into possible nutrient acquisition strategies of phloem-restricted pathogens.

## Materials and methods

### Phytoplasma strain, plant materials, and graft inoculation

The PPT phytoplasma strain was sourced from infected potato crops grown in agricultural regions of Washington and Oregon (Munyaneza et al. 2007). Tomato plants (*Solanum lycopersicum* cv. ‘Moneymaker’) were used as the host species. Phytoplasma inoculation was performed using the wedge-grafting technique, following previously described protocols (Wei et al. 2013). Control plants were mock-grafted using scions taken from healthy tomato plants. All analyses were conducted using fully expanded leaves harvested from the upper canopy 30 d after inoculation. Plants were maintained in a greenhouse at 25 °C under a 16-h light/8-h dark cycle.

### Chemical inhibition of autophagy

To assess the role of autophagy during infection, young branches from PPT phytoplasma-infected plants were excised and incubated upright in 1/10 Murashige and Skoog (MS) medium supplemented with 5 mM 3-methyladenine (3-MA) for up to 5 d. Samples for confocal microscopy and TEM were collected after 3 d, whereas samples for DNA extraction and phytoplasma titer measurement were collected after 5 d.

### DNA extraction and quantitative PCR for phytoplasma titer determination

Total DNA was extracted from tomato leaves using a modified CTAB method (Wei et al. 2004). Quantitative PCR (qPCR) was performed using an AriaMx Real-Time PCR System (Agilent Technologies, Santa Clara, California, United States) with Brilliant SYBR Green qPCR Master Mix (Agilent Technologies, Santa Clara, California, United States). Primers used for quantitation of phytoplasma 16S rRNA and the tomato actin reference genes are listed in Table S1. The phytoplasma 16S rRNA gene was quantified using primers MPPLPPT16SF2/MPPLPPT16SR2 (Wu et al. 2012), whereas the tomato *Actin* gene served as an internal reference and was quantified with SlActinF/SlActinR (Inaba et al. 2023). Melt-curve analysis confirmed the specificity of each amplicon by showing a single peak.

### Protein extraction and immunoblotting

#### ATG8 and ATG8-phosphatidylethanolamine detection

Leaf tissues (100 mg) from mock and PPT phytoplasma-infected plants were frozen and ground in liquid nitrogen. Proteins were extracted in lysis buffer (50 mM Tris-HCl, pH 7.5, 150 mM NaCl, 1 mM EDTA, 1% SDS, and protease inhibitor cocktail). Proteins were separated by SDS-PAGE under 2 conditions, including (i) standard gels, no urea, to resolve total ATG8 and (ii) 6 M urea gels to separate lipidated ATG8-phosphatidylethanolamine (ATG8-PE) from nonconjugated ATG8. Proteins were transferred to PVDF membranes (MilliporeSigma, Burlington, Massachusetts, United States), blocked with 5% nonfat milk in TBS-T, then probed with anti-ATG8 (Agrisera AB, Vännäs, Sweden) overnight at 4 °C. Alkaline phosphatase-conjugated anti-rabbit IgG secondary antibodies and BCIP/NBT color development (Promega, Madison, Wisconsin, United States) were used for detection.

#### PPT-lipase detection

Total proteins were extracted from leaves of PPT phytoplasma-infected and mock control tomato plants as described by Inaba and Nagy (2018). Proteins were separated on a Novex WedgeWell 4% to 20% Tris-Glycine gel (Invitrogen, Thermo Fisher Scientific, Carlsbad, California, United States), then transferred to a PVDF membrane (MilliporeSigma, Burlington, Massachusetts, United States) in Tris-Glycine transfer buffer (25 mM Tris, 192 mM glycine, 0.1% SDS) at 400 mA for 90 min. Membranes were blocked in TBS-T with 5% nonfat dry milk for 1 h at room temperature, then incubated with anti-PPT-lipase primary antibody (custom polyclonal, see below) overnight at 4 °C. After three 10 min washes in TBS-T, membranes were incubated with anti-rabbit IgG conjugated to alkaline phosphatase (Promega, Madison, Wisconsin, United States) for 1 h at room temperature, and bands were visualized with BCIP/NBT chromogenic substrate (Thermo Fisher Scientific, Waltham, Massachusetts, United States) according to the manufacturer's instructions. An antibody targeting the immunodominant protein (IDP) of PPT phytoplasma (PPT-IDP) (Inaba et al. 2023) was used as the internal control.

### Confocal imaging of autophagy markers, ER, phytoplasma, and neutral lipids

Paraffin-embedded stem sections (10 µm) were prepared from mock and PPT-infected plants. For 3-MA treatment experiments, sections were taken after 3 d of branch incubation. Stems were fixed in 4% paraformaldehyde, embedded in paraffin, sectioned on a Leitz-1512 rotary microtome (Ernst Leitz Wetzlar GmbH, Wetzlar, Germany), and deparaffinized (Inaba et al. 2023). After blocking (3% BSA, 0.1% Triton X-100), sections were incubated with the following primary antibodies: mouse anti-HDEL (ER marker; Invitrogen, Thermo Fisher Scientific, Carlsbad, California, United States), rabbit anti-PPT-IDP (Inaba et al. 2023), rabbit anti-ATG8, and rabbit anti-ATG12 (Agrisera AB, Vännäs, Sweden). Alexa Fluor 405- or 488-conjugated anti-mouse and anti-rabbit secondary antibodies (Thermo Fisher Scientific, Waltham, Massachusetts, United States, and Abcam, Cambridge, United Kingdom, respectively) were used for detection. Neutral lipids were stained with BODIPY 493/503 (1 µg mL^−1^; 1 h; excitation 488 nm). Images were acquired on a Zeiss LSM710 confocal microscope (Carl Zeiss, Jena, Germany) using identical laser/detector settings across treatments. Z-stacks (40 to 150 optical sections) were processed in Zeiss Zen 2012 Pro (Carl Zeiss, Jena, Germany) to generate maximum-intensity projections.

### Transmission electron microscopy

Stem tissues from mock, infected, and 3-MA–treated plants (3 d) were fixed and processed as described by Wei et al. (2022). Ultrathin sections were imaged on a Hitachi HT-7700 TEM (Hitachi High-Technologies Corp., Tokyo, Japan) at 80 kV. For general ultrastructure (chloroplast integrity, ER aggregates, and LDs), standard uranyl acetate/lead citrate staining was applied (Wei et al. 2022).

### Blue native PAGE of thylakoid complexes

Thylakoid membrane complexes were analyzed by blue native PAGE (BN-PAGE) with minor modifications of published protocols (Cui et al. 2011; Rantala et al. 2018). Tomato leaves were homogenized in extraction buffer (50 mM HEPES–KOH, pH 7.5, 330 mM sorbitol, 2 mM EDTA, 1 mM MgCl_2_, 5 mM ascorbate, 0.05% BSA, protease inhibitors) and filtered through 4 layers of Kimwipes. After centrifugation (2,500 × *g*, 5 min, 4 °C), pellets were washed with Buffer I (50 mM HEPES–KOH, pH 7.5, 5 mM sorbitol) and Buffer II (50 mM HEPES–KOH, pH 7.5, 100 mM sorbitol, 10 mM MgCl_2_). Thylakoids were solubilized in ACA buffer (750 mM ε-aminocaproic acid, 50 mM Bis–Tris, pH 7.0, 0.5 mM EDTA) with 2% digitonin (10 min, RT). After centrifugation (18,000 × *g*, 15 min, 4 °C), supernatants were mixed with native sample buffer (500 mM ε-aminocaproic acid, 5% Coomassie G-250) and run on Native PAGE Novex 3% to 12% Bis–Tris gels (Invitrogen, Carlsbad, California, United States).

### Lipid analyses

#### Polar lipids by liquid chromatography–tandem mass spectrometry

Polar lipids were profiled in upper leaves collected 3 months after graft inoculation from infected and mock plants. Approximately 0.1 g symptomatic tissue was incubated with 0.6 mL isopropanol containing 0.01% butylated hydroxytoluene at 75 °C for 15 min, followed by extraction with chloroform/methanol/water (30:41.5:3.5, v/v/v) for 36 h at room temperature. Organic solvents were evaporated under N_2_, and dried extracts were analyzed on a 4000 Q TRAP LC–MS/MS system (Kansas Lipidomics Research Center). A SPLASH Lipidomix standard (Avanti Polar Lipids) was used for quantification. Data were acquired from 5 biological replicates per condition. MGDG/DGDG ratios and phospholipid class totals were computed from internal-standard–normalized values. Experiments were performed with 5 independent biological replicates. Data are presented as mean ± SD. Statistical significance was assessed by *t*-test in Microsoft excel; *P* < 0.05 was considered significant.

#### Neutral lipids by thin-layer chromatography

Plant lipids were extracted as in the lipidomic protocol used in this study, then applied to thin-layer chromatography (TLC) Silica gel 60 plates (MilliporeSigma, Burlington, Massachusetts, United States), and developed in hexane: diethyl ether: acetic acid, 70:30:1, v/v/v. Neutral lipids were visualized with 0.03% Coomassie Brilliant Blue R-250 in 20% methanol and destained in 20% methanol. The lipid standards, including a TAG standard (Pointe Scientific, Inc., Canton, Michigan, United States), 1,3-dipalmitin (DAG, Toronto Research chemicals, Vaughan, ON, Canada), and 1-stearoyl-rac-glycerol (MAG, Toronto Research chemicals, Vaughan, ON, Canada) were used. TAG band intensity was quantified using the histogram function in Adobe Photoshop. At least 3 biological replicates were analyzed per condition.

### Gateway-based cloning and heterologous expression of PPT-lipase and yeast growth assay

The coding sequence of the PPT-lipase, along with a catalytic triad mutant (S99A/D208N/H242A), was prepared for heterologous expression using Gateway cloning technology. The PPT-lipase open reading frame was PCR-amplified with attB-flanked primers (Table S1), and the mutant version was synthesized commercially (Integrated DNA Technologies, Coralville, Iowa, United States). Entry clones were generated by BP recombination of the attB-flanked fragments with the donor vector Gateway pDONR/Zeo Vector using BP Clonase II (Thermo Fisher Scientific, Waltham, Massachusetts, United States). The resulting attL-containing entry clones were subsequently recombined into the galactose-inducible yeast expression vector pYES-DEST52 and the plant binary vector pGWB401 using LR Clonase II. Recombinant plasmids were selected on Zeocin (pDONR/Zeo), ampicillin (pYES-DEST52), or kanamycin (pGWB401). The pYES-DEST52 constructs were transformed into *Saccharomyces cerevisiae* strain INVSc1 (MATa his3Δ1 leu2 trp1-289 ura3-52) using the lithium acetate method, and transformants were selected on synthetic complete (SC) medium lacking uracil. For plant assays, pGWB401 constructs were introduced into *Agrobacterium tumefaciens* strain LBA4404 via electroporation for transient expression experiments.

For yeast growth assays, transformants were grown overnight in SC-Ura medium containing 2% glucose, harvested, washed, and adjusted to an optical density (OD_600_) of 0.5. Ten-fold serial dilutions were prepared, and 5 μL aliquots were spotted onto SC-Ura agar plates containing either 2% glucose or 2% galactose (inducing). Plates were incubated at 30 °C for 2 to 3 d, and growth was monitored by imaging.

### Bioinformatic identification and in silico analyses of PPT-lipase

Phytoplasma lipases were searched using the keyword “Phytoplasma lipases” in the NCBI database and then blasted against the PPT phytoplasma genome (JANHJP000000000). Signal peptides of the potential phytoplasma lipase were predicted with SignalP 6.0 (https://services.healthtech.dtu.dk/services/SignalP-6.0/). Protein structure models of PPT-lipase WT and the catalytic triad mutant were generated using SWISS-MODEL (https://swissmodel.expasy.org/), with the crystal structure of *Mycobacterium tuberculosis* MGL K74A (PDB ID: 7OZM) serving as the template (Grininger et al. 2021). Model quality was assessed using QMEANDisCo global scores (Studer et al. 2020). Sequence similarity and structural comparisons were conducted using the Protein Data Bank (PDB) (https://www.rcsb.org/). Multiple sequence alignments of PPT phytoplasma lipases and bacterial lipases were generated in BLASTP. Conservation of the catalytic triad (Ser–Asp–His) was assessed across homologs. The alignment of phytoplasma lipases was performed in Geneious Prime version 2026.02 using Clustal Omega.

### Production of polyclonal antibody against PPT phytoplasma lipase

A codon-optimized full-length PPT-lipase gene was synthesized as a gBlock (Integrated DNA Technologies, Coralville, Iowa, United States; Table S1) based on the original gene sequence (JANHJP000000000) to improve expression in *E. coli*. The gene was amplified using primers PPT-lipase-F-attB1 and PPT-lipase-R-attB2 (Table S1) and cloned into the pDONR221 entry vector using BP Clonase II (Thermo Fisher Scientific, Waltham, Massachusetts, United States). The coding sequence was subsequently transferred into the pDEST17 expression vector containing an N-terminal 6×His tag using LR Clonase II and transformed into *E. coli* LOBSTR-BL21(DE3)-RIL cells (Kerafast, Boston, Massachusetts, United States). Protein expression was induced with 1 mM IPTG, and cultures were incubated for 16 h at 18 °C. The recombinant protein was extracted and solubilized in 8 M urea prepared in sodium phosphate buffer at 4 °C. SDS-PAGE analysis showed a protein band of the expected size, which was excised and submitted to Pacific Immunology (Ramona, California, United States) for the production of rabbit polyclonal antibodies. The resulting antisera were used for subsequent experiments.

## Results

### Enhanced autophagy in PPT phytoplasma-infected tomato plants revealed by ATG8-PE accumulation

**Figure 1:**
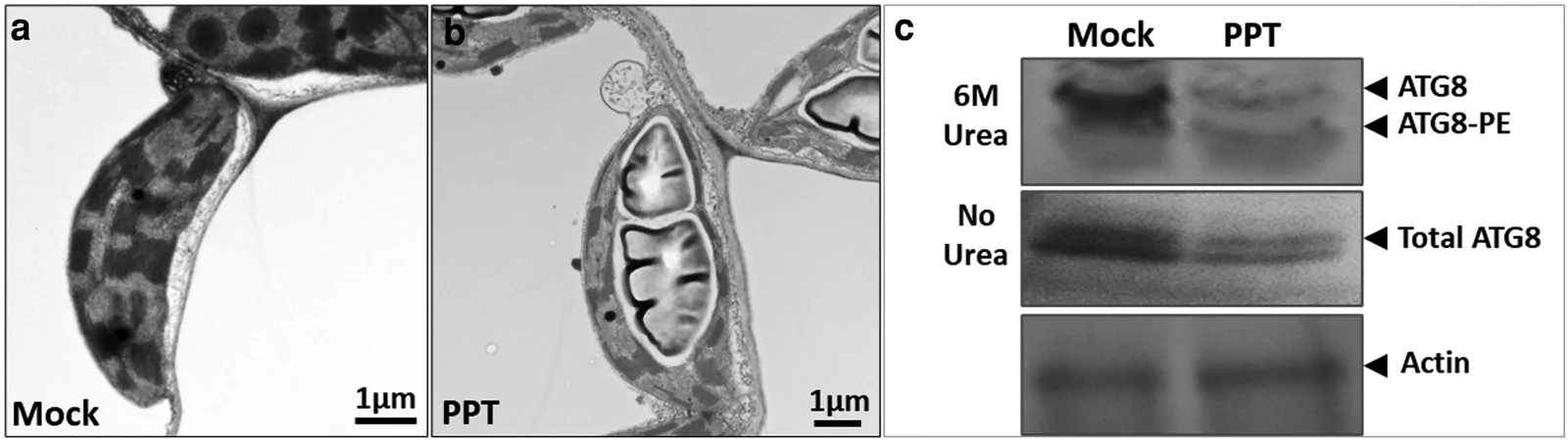
Activation of autophagy by PPT phytoplasma infection. a) Control tomato plants that were mock-inoculated displayed normal chloroplast structures. b) TEM revealed autophagosome-like structures adjacent to chloroplasts damaged by PPT phytoplasma infection, where extremely swollen starch grains were observed. c) Western blot analysis of tomato protein extracts confirmed the induction of autophagy during PPT infection. Proteins were separated by urea-SDS-PAGE, which enables the resolution of non-lipidated ATG8 from its lipidated form (ATG8-PE). The lipidation of ATG8 to ATG8-PE is a defining step in autophagosome formation and a hallmark of autophagy activation. PPT-infected plants exhibited a marked increase in ATG8-PE levels compared with mock controls, indicating enhanced autophagic activity. Actin was used as a loading control to verify equal protein loading across lanes. Scale bar = 1 um.

Autophagosome-like bodies were frequently observed near damaged chloroplasts in infected cells compared with mock controls (Fig. 1a and b), suggesting that autophagy was activated in response to organelle stress. This is consistent with our previous findings that PPT phytoplasma infection in tomato plants disrupts carbohydrate metabolism by impairing starch breakdown, leading to chloroplast injury (Wei et al. 2022).

Autophagy involves the lipidation of ATG8, which conjugates with phosphatidylethanolamine (PE) to associate with autophagic membranes (Martens and Fracchiolla 2020). The ratio of lipidated ATG8-PE to free ATG8 serves as an indicator of autophagic activity (Martens and Fracchiolla 2020; Huang et al. 2022). To assess whether PPT phytoplasma infection activates autophagy, western blot analysis was conducted using an ATG8-specific antibody on protein extracts from infected and mock control plants. Without urea, the lipidated ATG8-PE form was poorly resolved; however, the addition of 6 M urea enabled clear separation from nonconjugated ATG8. The results showed that PPT phytoplasma infection increased ATG8-PE levels while decreasing free ATG8 (Fig. 1c), indicating enhanced autophagic activity. These findings demonstrate that phytoplasma infection stimulates host autophagy.

### Confocal microscopy analysis confirms autophagy activation in response to phytoplasma infection

**Figure 2:**
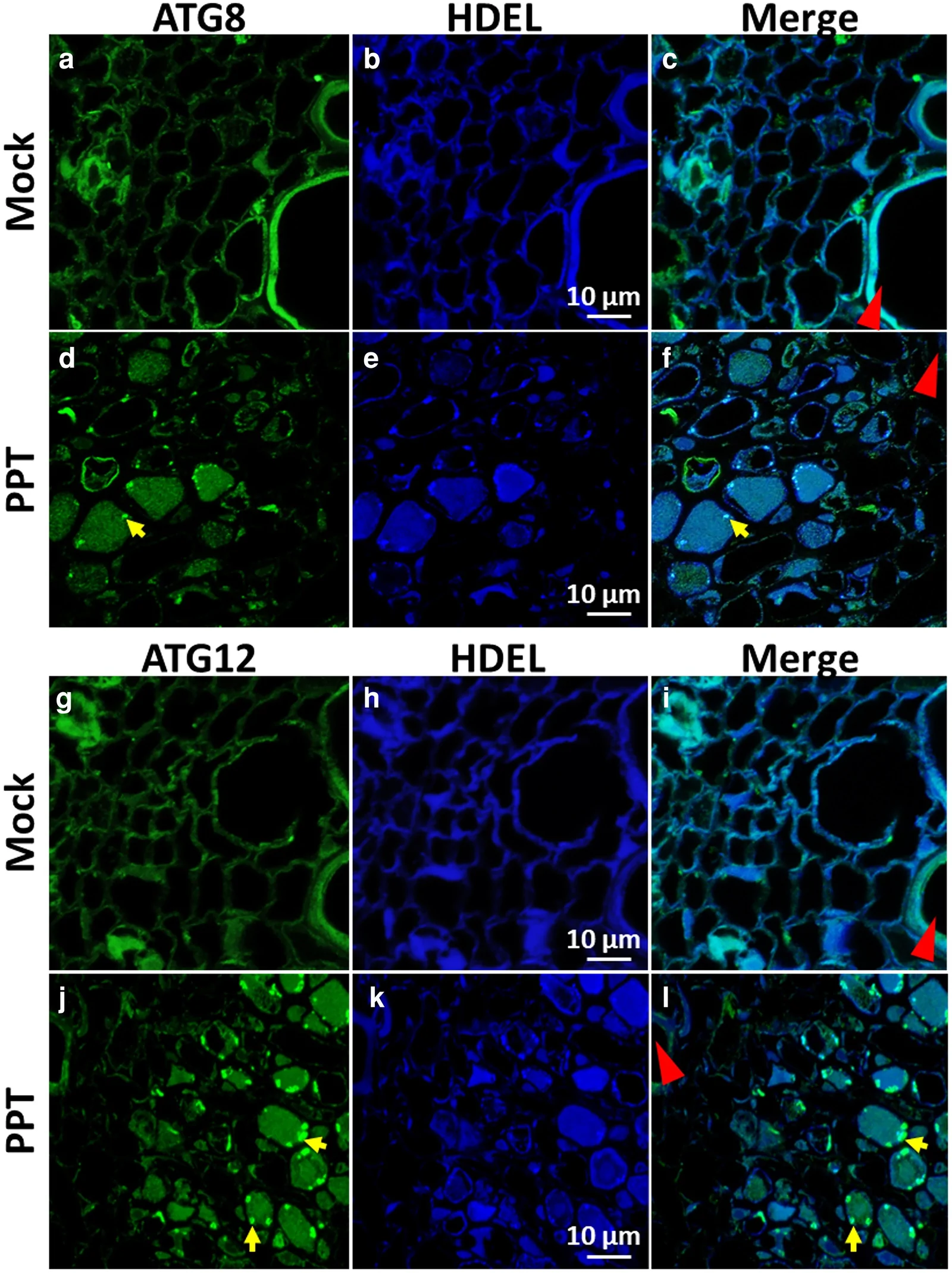
Confocal visualization of autophagy induction in tomato during PPT phytoplasma infection. Tomato stem sections were examined following PPT infection using immunofluorescence labeling of ATG8 and ATG12 with specific primary antibodies and green, fluorescent secondary antibodies, alongside mock-inoculated controls a, d, g, and j). The ER was labeled with an anti-HDEL primary antibody and a blue, fluorescent secondary antibody b, e, h, and k). Merged images c, f, i, and l) combine the 2 channels shown to the left (eg, panel c = a + b). Punctate ATG8- and ATG12-positive structures are indicated by yellow arrows, which correspond to autophagosomes that were rarely detected in mock controls. The presence of ATG8- and ATG12-positive puncta indicates autophagy activation, and their partial colocalization with ER labeling suggests the ER may serve as a membrane source during autophagosome formation. The red triangles indicate xylem regions. Scale bar = 10 μm.

Confocal microscopy was used to visualize autophagy in stem cross-sections from PPT phytoplasma-infected and mock controls (Fig. 2). Samples were immunostained with primary antibodies against ATG8 and ATG12, key proteins involved in autophagosome formation, and then incubated with fluorescent secondary antibodies. ATG8 labels autophagic membranes, while ATG12 forms a complex with ATG5 and associates with ATG16 to facilitate early membrane expansion in autophagosome formation (Iriondo et al. 2023). As autophagy progresses, small punctate structures (autophagosomes) appear in the cytoplasm, serving as indicators of autophagic activity (Schüssele et al. 2023). In control plants, fluorescence signals for ATG8 and ATG12 were faint and diffusely distributed throughout the phloem cytosol, indicating only basic autophagic activity (Fig. 2a and g). In contrast, PPT phytoplasma-infected plants exhibited intense, punctate fluorescence for both proteins, indicating strong induction of autophagy. These puncta, small, distinct, dot-like fluorescent structures, representing localized accumulations of autophagy-related proteins, correspond to sites of autophagosome formation and serve as markers of active autophagy (Fig. 2d and j).

Our previous work demonstrated that phytoplasma infection induces ER stress, leading to disruption of the ER network as detected by anti-HDEL antibody staining (Inaba et al. 2023). In the current study, confocal imaging further revealed that ATG8- and ATG12-labeled puncta in infected tissues frequently overlapped with HDEL-positive ER aggregates (Fig. 2h and k), whereas such colocalization was rarely observed in mock controls (Fig. 2b and e). This spatial association indicates that autophagosomes preferentially form in regions of ER stress, highlighting the ER as a key platform for autophagy initiation (Fig. 2i and l). Infected samples also showed elevated levels of ATG8- and ATG12-positive puncta, consistent with the western blot data, confirming enhanced autophagic activity under phytoplasma infection conditions. These findings extend previous observations of ER stress by demonstrating its close spatial and functional connection to autophagosome formation.

### Lipid remodeling under phytoplasma infection

**Figure 3:**
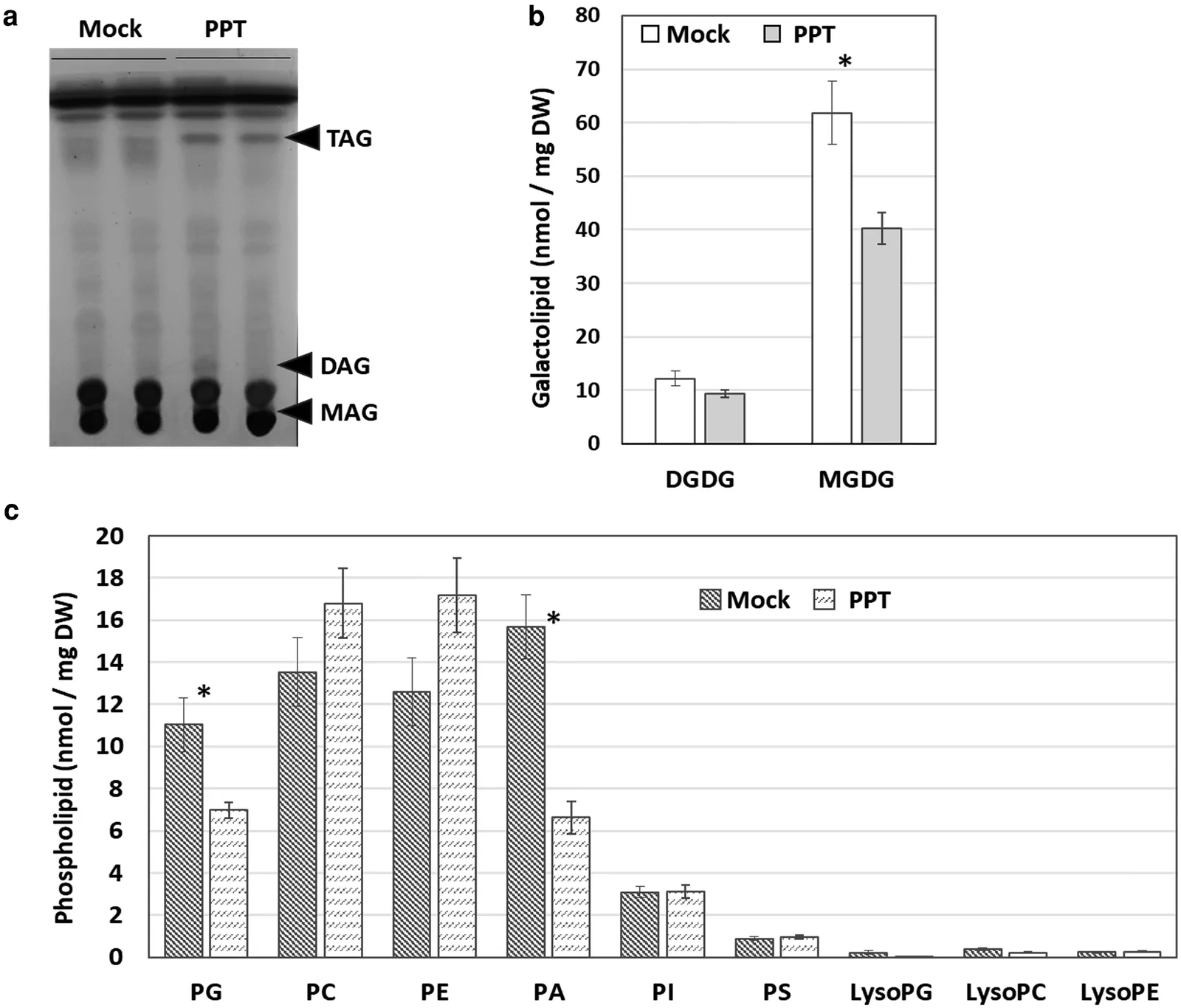
TAG and polar lipid alterations in tomato leaves during PPT phytoplasma infection. a) TAG accumulation. Total lipids were extracted from young upper leaves of PPT-infected and mock-inoculated tomato plants (3 months post-inoculation), separated by TLC, and visualized with Coomassie brilliant blue G-250 staining. PPT phytoplasma infection led to a strong increase in TAG compared to mock controls. b and c) Polar lipid composition. Polar lipids were also quantified from leaf samples at the same infection stage for TAG analysis by LC–MS/MS. b) Galactolipids, including MGDG and DGDG, were significantly altered, with PPT infection reducing the MGDG/DGDG ratio, indicative of thylakoid membrane remodeling. DW, dry weight. c) Phospholipids and lysophospholipids were differentially affected. Compared with mock control plants, PG and PA decreased significantly, PI and PS remained unchanged, while PC and PE increased in infected plants. LysoPG and LysoC declined, whereas LysoPE remained unchanged following infection. These shifts reflect infection-induced remodeling of both structural membrane lipids and lipid turnover. Values represent absolute amounts (nmol lipid per mg dry weight). Error bars show the standard error of the mean. Asterisks (*) indicate statistically significant differences relative to mock controls (Student's *t*-test, *P* ≤ 0.05).

To evaluate whether phytoplasma infection alters lipid metabolism, both neutral and polar lipid fractions were analyzed. TLC assay of neutral lipid fractions revealed an obvious accumulation of TAG in PPT phytoplasma-infected tomato plants compared with mock controls (Fig. 3a; Table S2). TAG is a major storage lipid that typically increases under stress conditions when membrane lipids are degraded, and fatty acids are redirected into storage pools (Yang and Benning 2018). Since phytoplasmas cannot be cultured independently and depend on host-derived nutrients, the elevated TAG levels suggest a reallocation of host carbon to storage lipids, which may provide an exploitable nutrient reservoir for the pathogen.

Polar lipid profiling by liquid chromatography–tandem mass spectrometry (LC–MS/MS) uncovered extensive remodeling of polar membrane lipids in PPT phytoplasma-infected tomato tissues (Fig. 3b and c). The chloroplast galactolipid monogalactosyldiacylglycerol (MGDG) declined markedly from 61.8 to 40.3 nmol mg^−1^ dry weight (DW), whereas digalactosyldiacylglycerol (DGDG) remained unchanged (Fig. 3b). This selective reduction decreased the MGDG/DGDG ratio, an indicator of thylakoid membrane organization and photosystem stability. The decline in MGDG, together with a reduction in phosphatidylglycerol (PG), another chloroplast-associated phospholipid (Fig. 3c), suggests chloroplast membrane degradation and impaired lipid synthesis, consistent with autophagy-mediated organelle turnover under infection-induced stress. Protein analyses by BN-PAGE and SDS-PAGE supported these lipidomic findings, showing that PPT phytoplasma infection disrupted thylakoid membrane organization and reduced Rubisco abundance, indicative of compromised chloroplast integrity and diminished photosynthetic function in infected tomato plants (Figure S1). Levels of phosphatidic acid (PA) were also significantly reduced in infected samples (Fig. 3c). This indicates that the pathogen actively interferes with the host's lipid-mediated signaling pathways, potentially suppressing PA-dependent immune responses. Since PA is a key secondary messenger in plant defense signaling (Park et al. 2004), its reduction suggests that the phytoplasma may subvert or suppress host defense mechanisms to facilitate successful colonization.

In infected tomato leaves, levels of phosphatidylcholine (PC) and PE showed an upward trend (Fig. 3c). The increase in these key ER-derived phospholipids indicates enhanced ER membrane production or restructuring, likely in response to ER stress and autophagosome development. Conversely, levels of phosphatidylserine (PS) and phosphatidylinositol (PI) remained relatively stable (Fig. 3c), indicating that membrane identity and associated signaling lipids were largely maintained. Additionally, lipidomic analysis revealed a decrease in lysophosphatidylglycerol (lysoPG) and lysophosphatidylcholine (lysoPC) in infected plants, while lysophosphatidylethanolamine (lysoPE) levels remained unchanged (Fig. 3c). The observed decline in lysophospholipids, coupled with increased PC and PE, implies a metabolic shift toward membrane remodeling and ER expansion, supporting ongoing autophagosome formation during infection.

### LDs are distributed around phytoplasma cells

**Figure 4:**
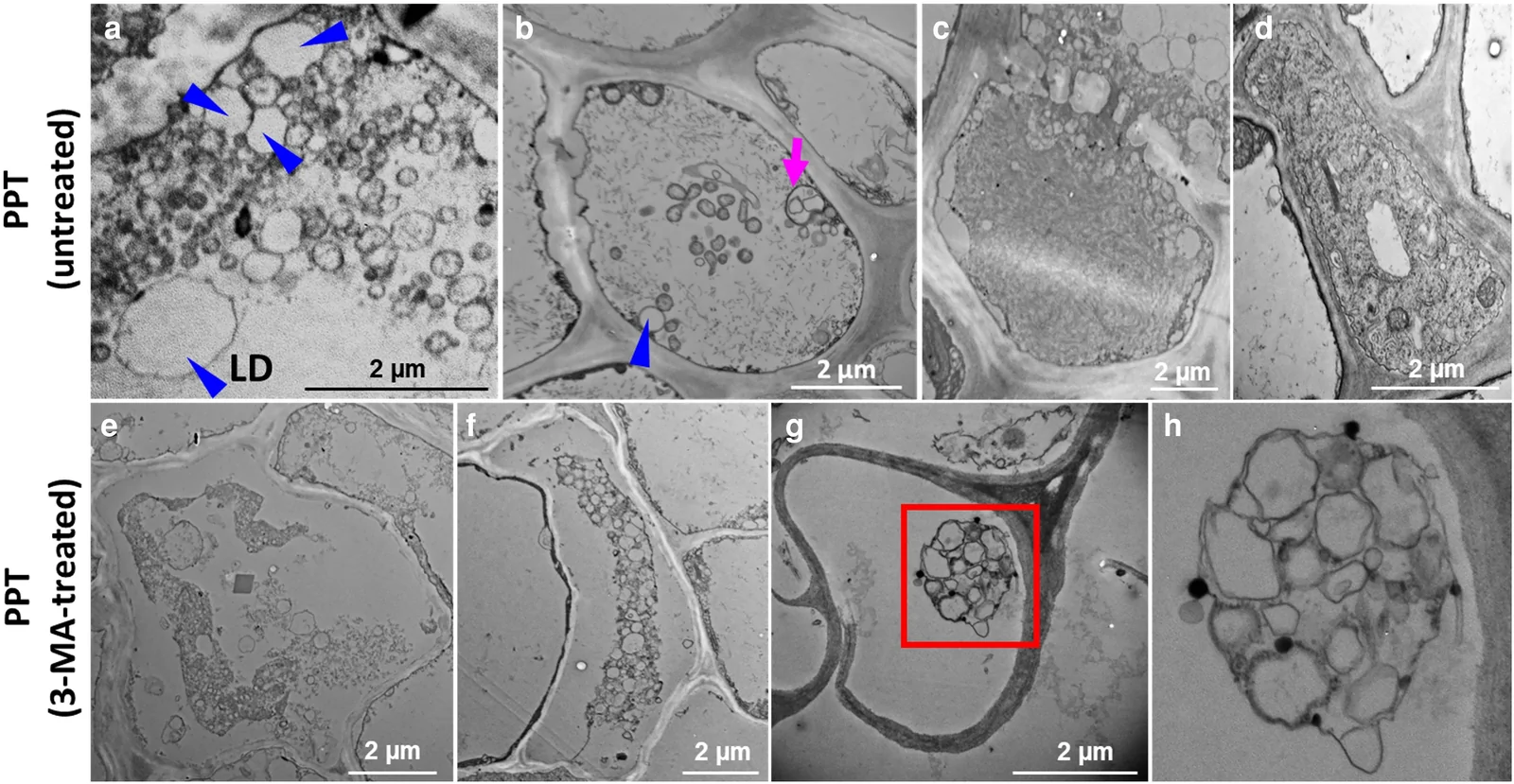
Visualization of LDs and protein aggregates in tomato plants infected with PPT phytoplasma using TEM. a to d) PPT phytoplasma-infected tomato plants without 3-MA treatment. a) Numerous LDs (indicated by blue arrowheads) are observed in close proximity to phytoplasma cells within sieve elements. The LDs vary in size and morphology. b) LDs, phytoplasma cells, and autophagosome-like bodies (pink arrow) engulfing cellular materials are visible within an infected sieve element. Some phytoplasma cells show elongation and division. Scattered phloem proteins (P-proteins) are also present throughout the sieve element. c and d) Protein aggregates induced by PPT phytoplasma infection are shown, enriched in P-proteins. In d), a stacked ER structure is also evident. e to h) PPT phytoplasma-infected tomato plants were treated with 3-MA, an autophagy inhibitor. e and f) Phytoplasma cells are rarely observed, and the abundance of protein aggregates is reduced compared with untreated infected plants. However, LDs appear enlarged and/or increased in number. g) Enlarged LDs are frequently clustered, consistent with abnormal accumulation caused by blocked autophagy. h) Close-up of the red boxed region in g), highlighting LD aggregation. These results suggest that PPT phytoplasma infection induces the formation of LDs, protein aggregates, and autophagosome-like structures in tomato sieve elements, while chemical inhibition of autophagy with 3-MA leads to abnormal LD accumulation and reduced protein aggregate formation. Scale bar = 2 μm.

To investigate the cellular manifestations of lipid remodeling during PPT phytoplasma infection, infected tomato leaf tissues were analyzed using transmission electron microscopy (TEM) and confocal microscopy. TEM analysis revealed numerous LDs within the sieve elements of phytoplasma-infected leaves (Fig. 4a and b). These LDs appeared as electron-translucent, round to irregular structures that displayed considerable variation in size and shape (Fig. 4a and b). LD size ranged from 0.3 to 1.2 µm. Many LDs were located adjacent to phytoplasma cells (Fig. 4a), a spatial arrangement not observed in control plants (Wei et al. 2022; Inaba et al. 2023). The abundance and proximity of LDs to phytoplasmas indicate that these lipids are actively produced or mobilized during infection, suggesting a dynamic involvement of lipid metabolism in host–pathogen interactions.

**Figure 5:**
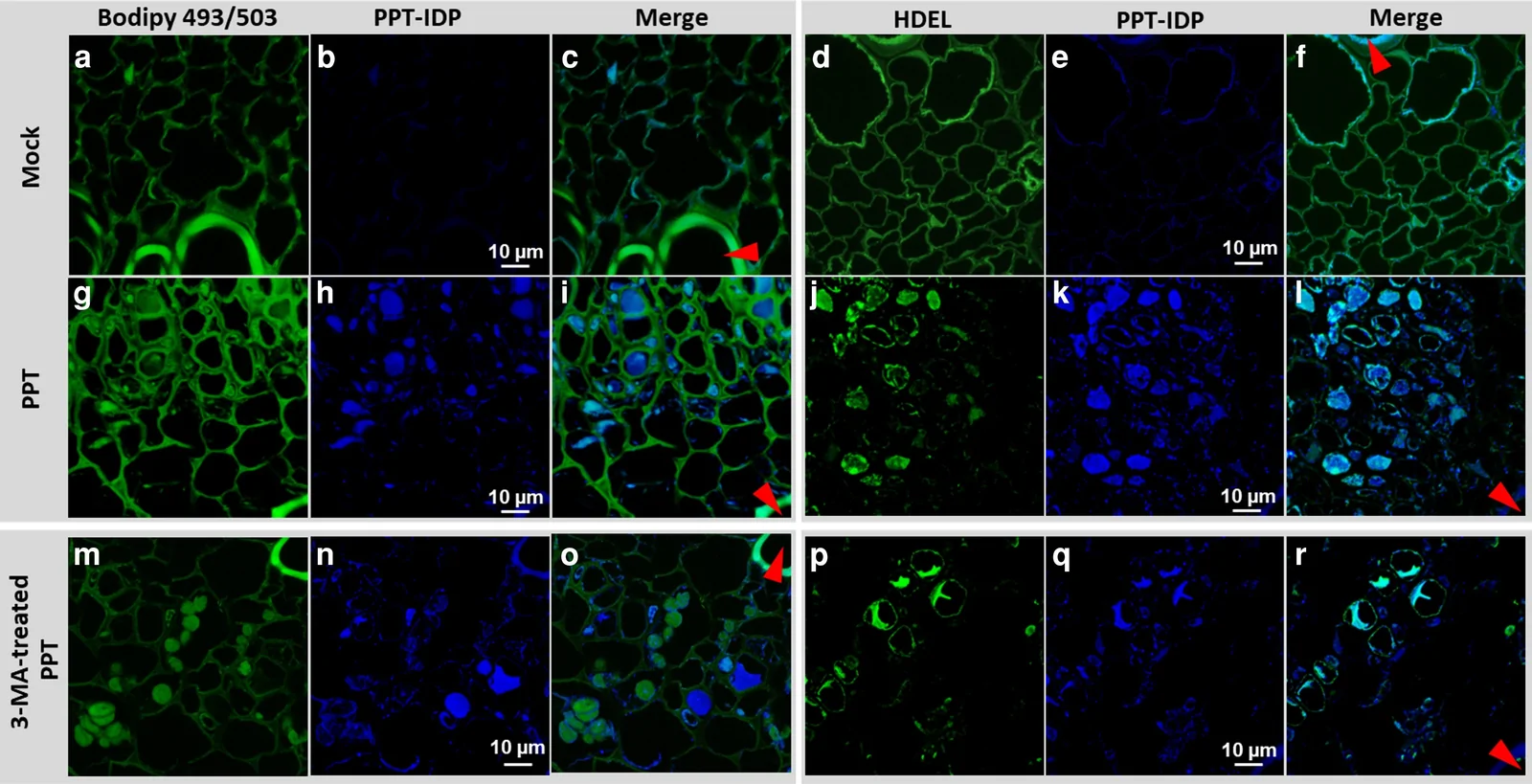
Confocal visualization of PPT phytoplasmas, LDs, and the ER network in tomato leaves after 3-MA treatment. a to f) Mock-inoculated control plants. g to l) PPT phytoplasma-infected plants without the autophagy inhibitor 3-MA treatment. m to r) PPT phytoplasma-infected plants treated with 3-MA. Phytoplasma cells were detected with anti-PPT-IDP primary antibody and Alexa Fluor405–conjugated secondary antibody (blue). Neutral lipids (TAG-containing LDs) were visualized using BODIPY 493/503 staining (green). The ER network was labeled with an anti-HDEL primary antibody and Alexa Fluor 488–conjugated secondary antibody (green). Merged images c, i, o, f, l, and r) combine the 2 channels shown to the left (eg, panel c = a + b). In PPT phytoplasma-infected plants without 3-MA treatment, partial colocalization of LDs g) and phytoplasma cells h) was observed (merge, i), and BODIPY 493/503 signals were more intense than in mock controls, indicating increased TAG accumulation. Following 3-MA treatment, phytoplasma signals were markedly reduced n) and LDs clustered into ball-like structures m and o) with limited colocalization with phytoplasma cells. PPT phytoplasma infection also induced ER aggregation j), but 3-MA treatment reduced both ER aggregate signals p) and phytoplasma presence q), with partial colocalization still detected in merged images r). The red triangles indicate xylem regions. Scale bar = 10 μm.

Confocal microscopy further confirmed PPT phytoplasma infection-associated alterations in LD distribution. Neutral lipids were visualized with BODIPY 493/503, phytoplasma cells were identified using an antibody targeting the IDP, and the ER resident protein or network was labeled with an antibody of HDEL (ER retention signal). In mock control plants, BODIPY fluorescence was primarily confined to cellular membranes (Fig. 5a), consistent with LDs being closely associated with the ER network under normal conditions (Fan et al. 2011). In contrast, infected tomato plants showed a clear redistribution of LDs; although some remained membrane-associated, a substantial proportion of BODIPY-positive structures colocalized with phytoplasma cells (Fig. 5g, h, and i). LDs were frequently observed encircling or partially surrounding phytoplasma bodies (Fig. 5i), indicating that the pathogen may attract or manipulate LD positioning. This spatial association suggests a potential functional interaction in which LDs, enriched in TAGs, may act as localized nutrient sources exploited by PPT phytoplasmas during infection.

### Autophagy supports phytoplasma survival

**Figure 6:**
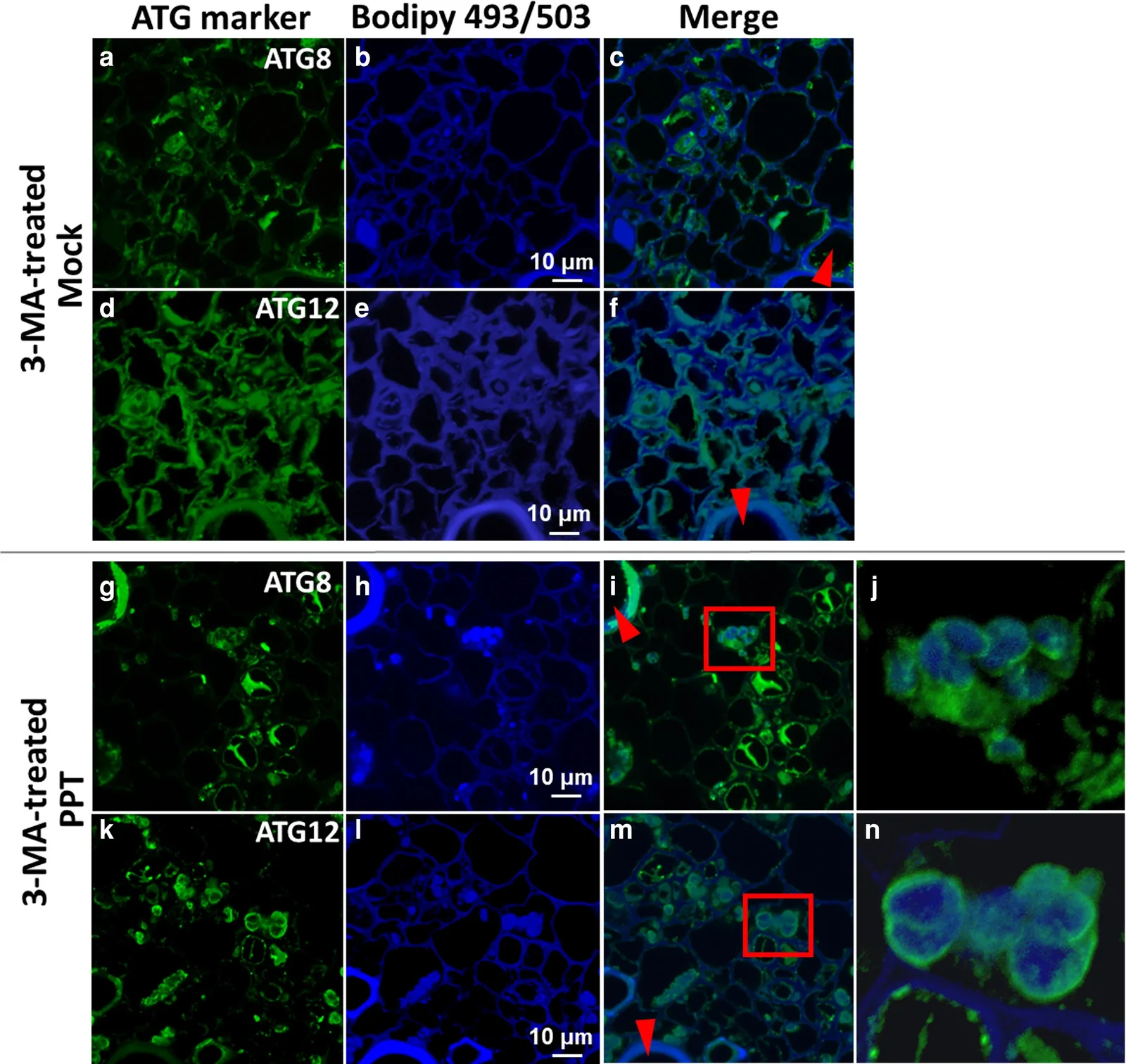
Confocal microscopy visualization of LDs and autophagy inhibition in tomato leaves following MA treatment. Neutral lipids were stained with BODIPY 493/503 (blue), and autophagy was detected by immunofluorescence labeling of ATG8 and ATG12 using primary antibodies and green, fluorescent secondary antibodies. a to f) 3-MA–treated, mock-inoculated plants. Neutral lipids a and d) appeared dispersed across the cytoplasm, while ATG8 b) and ATG12 e) signals revealed basal puncta formation. Merged images c and f) show partial overlap, indicating low-level autophagy despite chemical inhibition. g to n) 3-MA–treated PPT-infected plants. Neutral lipids g and k) accumulated abnormally, forming enlarged ball-like structures. ATG8 h) and ATG12 l) signals were markedly reduced compared with mock plants. Merged images i and m) and magnified insets of the red boxes j and n) highlight abnormal LD accumulation and clustered autophagy puncta associated with undegraded cellular materials. These features are consistent with blocked autophagic flux under 3-MA treatment in PPT-infected cells. The red triangles indicate xylem regions. Scale bar = 10 μm.

To investigate whether phytoplasmas activate and exploit host autophagy, infected tomato plants were treated with the autophagy inhibitor 3-MA. In healthy, unstressed plants, autophagy often functions at a basal maintenance level. Accordingly, both mock controls with and without 3-MA treatment exhibited diffuse ATG8 and ATG12 fluorescence with no discernible puncta (Figs. 2a and g and 6a and d), indicating minimal autophagosome formation under normal conditions. In contrast, PPT phytoplasma-infected plants without 3-MA treatment displayed numerous ATG8- and ATG12-positive puncta (Fig. 2d and j), reflecting strong autophagic activation during infection. Treatment with 3-MA markedly reduced the number of these puncta in infected tissues (Fig. 6g and k), confirming effective suppression of autophagy. Following 3-MA treatment, compared to untreated infected plants (Fig. 5g-l), phytoplasma fluorescence signals were substantially diminished (Fig. 5n), and LDs coalesced into large, spherical clusters (Figs. 5m and 6g, k, i, m, j, and n) that showed rare colocalization with phytoplasma cells (Fig. 5o). PPT infection normally induced prominent ER aggregation (Fig. 5j to l; Inaba et al. 2023), whereas 3-MA treatment led to a significant reduction in both phytoplasma abundance (Fig. 5n and q) and ER aggregate signals (Fig. 5p). Partial colocalization between the remaining ER structures and phytoplasmas was still detectable but it was limited (Fig. 5r).

TEM analysis corroborated the confocal observations, revealing distinct ultrastructural alterations in 3-MA–treated infected tissues (Fig. 4). ER-derived aggregates were narrower, less densely packed, and irregularly organized (Fig. 4e and f) compared with those in 3-MA–untreated infected plants (Fig. 4a to d). Many ER aggregates contained vesicle-like inclusions (Fig. 4e and f), reflecting disrupted membrane homeostasis. In the same tissues, large LD clusters accumulated within the sieve elements, forming compact spherical masses that were rarely associated with phytoplasma cells (Fig. 4g and h). Quantitative PCR confirmed that phytoplasma titer was significantly lower in 3-MA–treated infected plants than in untreated infected controls (Figure S2). These findings indicate that autophagy, rather than merely being a stress response, is probably exploited by phytoplasmas to boost their growth, likely by utilizing host membrane lipids and LDs as nutrient sources.

### Identification of a lipase in the PPT phytoplasma genome

The observation that LDs frequently colocalized with PPT phytoplasma cells (Figs. 4a and b and 5g to i), together with the evidence of increased TAG in infected plants (Fig. 3a), raised an important question of whether phytoplasmas may access or exploit host lipid resources to support their survival and proliferation. To explore this possibility, we conducted a genome-wide search for lipase-encoding genes. *Candidat*e phytoplasma lipases were first identified from the NCBI database and then queried against the PPT phytoplasma genome (JANHJP000000000; Wei et al. 2022). This analysis revealed a putative lipase (262 aa, 28.8 kDa), annotated as an alpha/beta hydrolase (WP_273585345.1), hereafter referred to as PPT-lipase, which contains the predicted catalytic triad characteristic of active lipases.

Protein structure prediction using the PDB indicated the structural similarity between PPT-lipase and monoacylglycerol lipases (MGLs) from humans and bacteria, such as *Palaeococcus ferrophilus* and *M. tuberculosis* (Table S3). MGLs hydrolyze monoacylglycerol (MAG) into free fatty acids and glycerol, which can serve as essential energy substrates (Jing et al. 2025). These findings suggest that PPT-lipase may possess lipolytic activity toward neutral lipid intermediates such as MAG.

**Figure 7:**
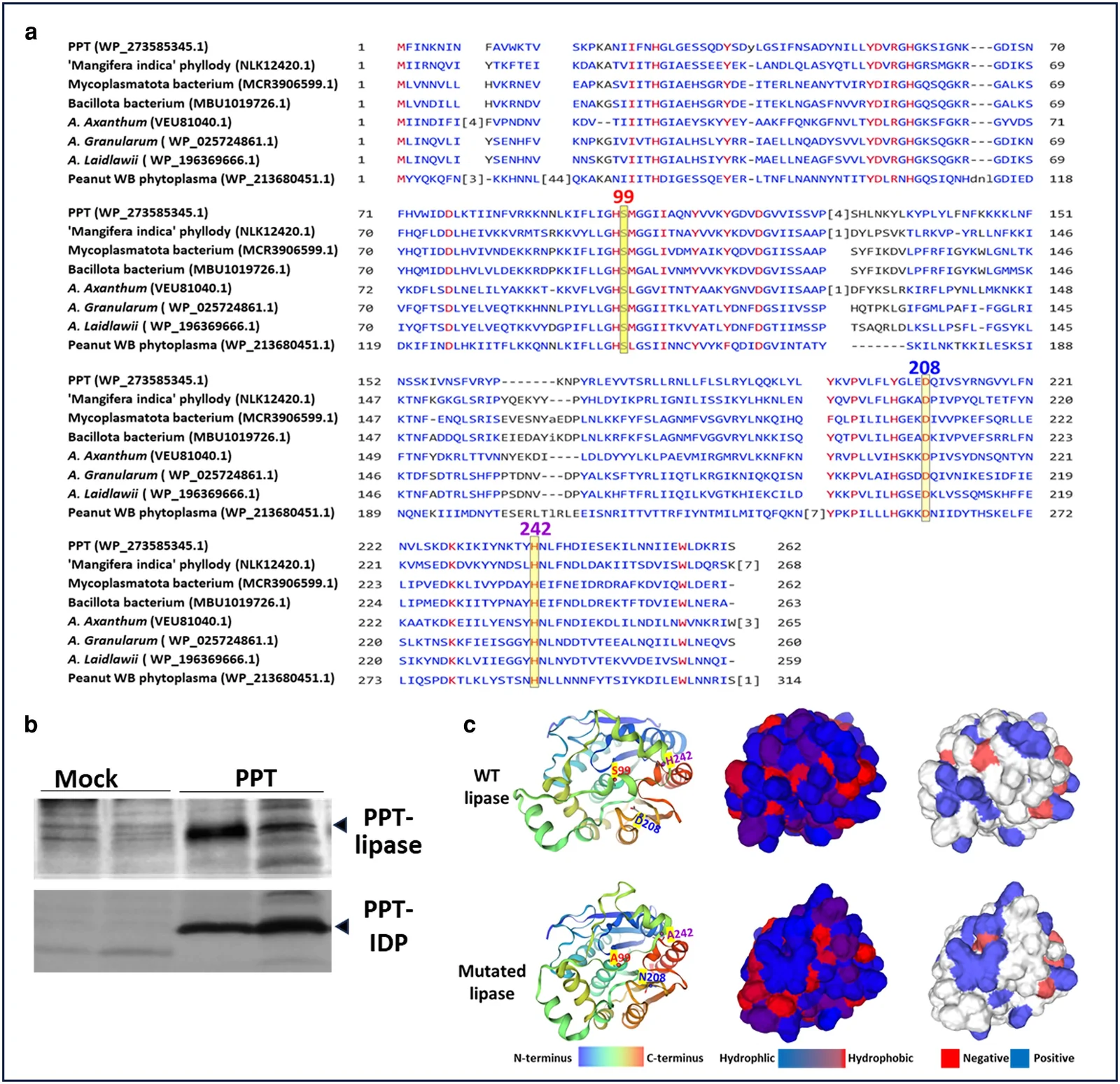
PPT phytoplasma encodes a MGL-like enzyme. a) Sequence alignment of PPT phytoplasma lipase (WP_273585345.1) with representative bacterial MGLs. Conserved residues are shown in red, and the catalytic triad (Ser99, Asp208, His242) essential for MGL activity (Karlsson et al. 1997) is highlighted in yellow. b) Western blot detection of PPT-lipase expression in tomato plants infected with PPT phytoplasma. Total proteins were extracted from infected and mock-inoculated (mock) leaves. Anti-PPT-IDP antibody detected a distinct band at the expected size in infected samples only, confirming the presence of PPT phytoplasma. Anti–PPT-lipase antibody revealed accumulation of PPT-lipase in infected plants but not in mock plants. c) Predicted 3D structures of WT and catalytic triad mutant PPT-lipase generated with SWISS-MODEL. Ribbon diagrams illustrate the conserved α/β-hydrolase fold, with the catalytic residues (Ser99, Asp208, His242) correctly aligned in the WT enzyme to form a functional active site. Substitution of these residues (S99A/D208N/H242A) disrupted active-site geometry, abolishing a coherent catalytic pocket. Electrostatic surface potential maps further revealed marked alterations in charge distribution around the mutant's active site, consistent with impaired substrate binding and reduced catalytic capacity. Both models exhibited robust structural quality; the WT and mutant models yielded QMEANDisCo global scores of 0.58 ± 0.05 and 0.60 ± 0.05, respectively, with 92.55% and 92.61% of residues falling in the most favored regions of the Ramachandran plot.

Further protein BLAST and alignment analyses revealed that lipase homologs are conserved across 57 phytoplasma strains (Figure S4). SignalP 6.0 analysis predicted secretory signal peptides in lipases from 12 phytoplasmas, including “*Candidatus* Phytoplasma fraxini” (WYY26227.1) and “*Candidatus* Phytoplasma stylosanthis” (WP_318676370.1, Table S4). No signal peptide was predicted for the PPT-lipase, implying that it is unlikely to be secreted via the classical Sec pathway and may instead function intracellularly within phytoplasma cells. Amino acid sequence alignment of the PPT phytoplasma lipase with other bacterial lipases showed the highly conserved residues, including the canonical catalytic triad consisting of Serine (S99), Aspartic acid (D208), and Histidine (H242; Fig. 7a), which are characteristic of catalytically active lipases (Karlsson et al. 1997).

### In planta PPT-lipase expression and localization

To verify PPT-lipase expression during infection, a polyclonal antibody was generated using a recombinant antigen derived from an optimized coding sequence, following unsuccessful attempts to express the native PPT-lipase in *Escherichia coli*. Total proteins extracted from mock-inoculated and PPT phytoplasma-infected plants were analyzed by western blotting using the anti-PPT-lipase antibody. A specific band of approximately 28.8 kDa was detected in infected plant samples, while no signal was observed in the mock controls (Fig. 7b). These results confirm that PPT-lipase is produced in planta during phytoplasma infection.

Confocal imaging further supported the western blot results. PPT-lipase fluorescence signals were immunodetected in infected tissues and were frequently observed in close proximity to host ER structures labeled with the anti-HDEL antibody, while no signals were detected in mock controls (Figure S3a). PPT-lipase fluorescence was also partially colocalized with neutral lipids detected by BODIPY 493/503 (Figure S3b). These results indicate that PPT-lipase is expressed during infection and localizes near host ER structures and lipid-rich regions, consistent with the spatial distribution of phytoplasma IDP signals (Fig. 5j to l).

### PPT-lipase activity enhances yeast growth

**Figure 8:**
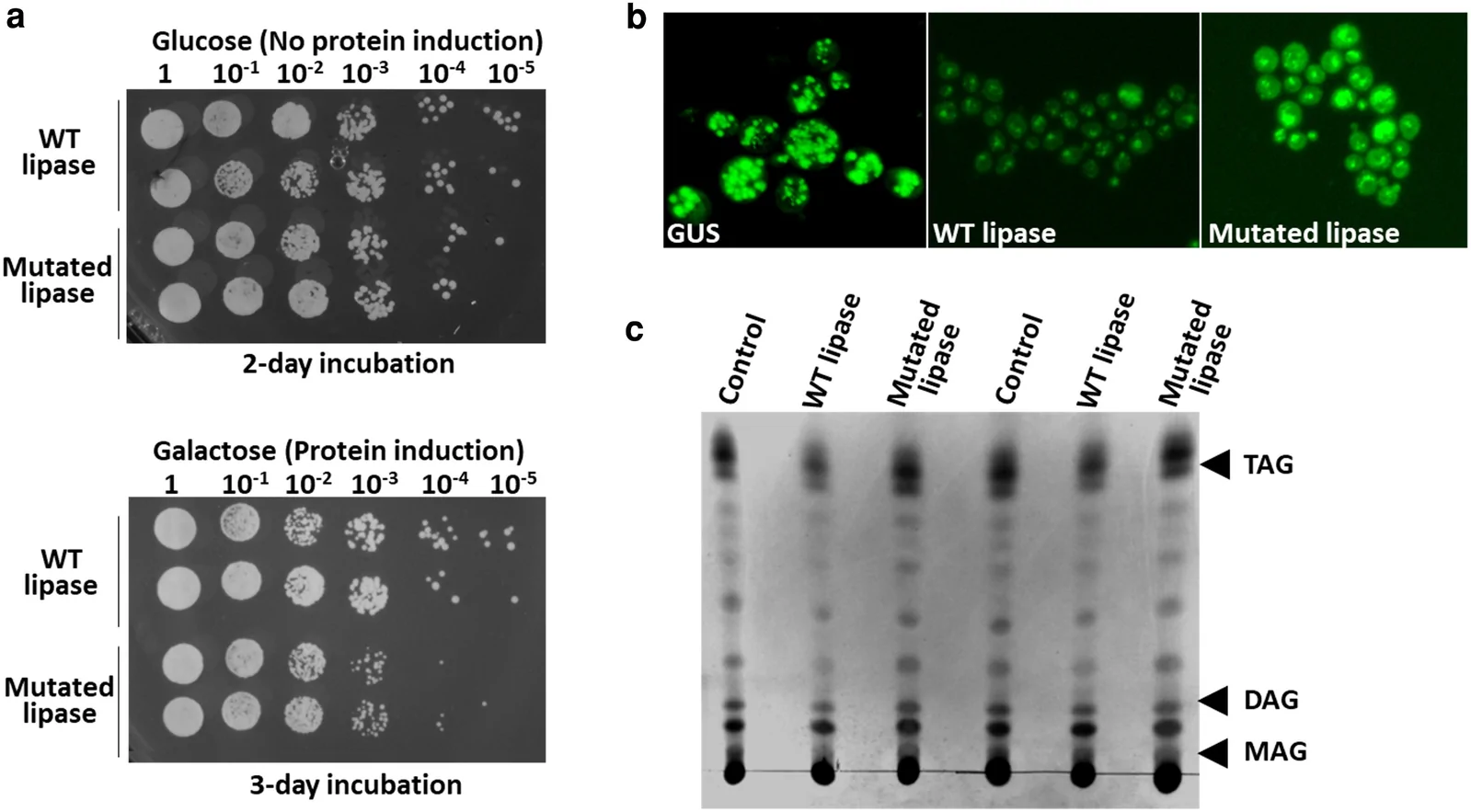
PPT-lipase overexpression reduces neutral lipid level levels in yeast and *N. benthamiana*. a) Yeast growth assay. WT and catalytic triad mutant (S99A/D208N/H242A) PPT-lipases were cloned into the galactose-inducible yeast expression vector pYES-DEST52. On glucose medium, the GAL promoter was repressed, and both WT and mutant PPT-lipase transformants grew normally. On galactose medium, induction of WT PPT-lipase enhanced yeast growth across serial dilutions, whereas the catalytic triad mutant showed reduced growth. These results indicate that the catalytic activity of PPT-lipase promotes yeast growth, likely through TAG hydrolysis and mobilization of lipid-derived energy. b) Confocal microscopy of yeast cells overexpressing WT or mutated PPT-lipase, with LDs stained using BODIPY 493/503. WT PPT-lipase–expressing cells showed reduced LD fluorescence intensity, indicating TAG hydrolysis and mobilization. In contrast, the catalytic triad mutant exhibited stronger fluorescence with larger and more abundant LDs, consistent with impaired lipase activity and TAG accumulation. (c) Heterologous expression in *N. benthamiana*. WT and mutant PPT-lipase constructs were cloned into a plant expression vector, transformed into *A. tumefaciens* strain LBA4404, and transiently expressed in *N. benthamiana* leaves by agroinfiltration. Total neutral lipids were extracted from infiltrated leaves, separated by TLC. Overexpression of WT PPT-lipase led to reduced TAG levels compared with the mutant.

To evaluate the functional importance of the predicted catalytic residues of PPT-lipase, a catalytic triad mutant (S99A/D208N/H242A) was generated. Structural modeling using SWISS-MODEL predicted conformational alterations in the mutant enzyme relative to the wild type (WT), consistent with disruption of catalytic integrity (Fig. 7c). Enzymatic function was assessed heterologously in *S. cerevisiae* using a galactose-inducible pYES-DEST52 expression system. Under non-inducing (glucose) conditions, all strains exhibited comparable growth, indicating that basal expression of WT or mutant PPT-lipase did not significantly affect cell proliferation (Fig. 8a, top panel). Upon galactose induction, however, yeast expressing WT PPT-lipase displayed enhanced growth relative to cells expressing the catalytic mutant (Fig. 8a, bottom panel). This observation suggests that the catalytic activity of PPT-lipase may contribute to improved cellular fitness under inducing conditions and is consistent with a possible role in lipid metabolism.

Neutral lipid distribution was examined by BODIPY 493/503 staining and fluorescence microscopy. Yeast cells expressing β-glucuronidase (GUS) as a negative control or the catalytic mutant exhibited strong fluorescence and abundant LDs, indicating neutral lipid accumulation. In contrast, cells expressing WT PPT-lipase showed reduced fluorescence and fewer LDs (Fig. 8b), consistent with enhanced neutral lipid turnover.

WT and mutant constructs were also transiently expressed in *Nicotiana benthamiana* leaves, and lipid profiles were analyzed by TLC. Expression of WT PPT-lipase resulted in a shift in lipid composition compared to empty vector controls, characterized by reduced TAG levels (Fig. 8c; Table S2). Leaves expressing the catalytic mutants retained higher TAG levels similar to those of control samples. These findings are consistent with yeast-based assays and indicate that catalytic activity contributes to changes in neutral lipid turnover in plant tissue. Although PPT-lipase shares structural similarity with MGL-like enzymes and lacks a predicted secretion signal, its expression alters host neutral lipid homeostasis, supporting a potential role in modulating lipid dynamics during phytoplasma infection.

## Discussion

Phytoplasmas are obligate intracellular bacteria with highly reduced genomes, making them dependent on host metabolic pathways for survival and proliferation (Oshima et al. 2004; Tan et al. 2021). Their limited biosynthetic capacity forces them to exploit host cellular machinery, although the mechanisms governing this dependence remain poorly understood. Previous research has shown that phytoplasma infection induces ER stress and activates the UPR, leading to ER protein aggregation and disruption of ER structure (Inaba et al. 2023). This study further examines phytoplasma infection–induced activation of autophagy, lipid metabolic remodeling, and increased LD formation in host plants. A phytoplasma-derived lipase was also identified, and its potential role in accessing host lipids was evaluated.

Autophagy was activated during the PPT phytoplasma infection in tomato plants, as shown by increased ATG8-PE lipidation, the formation of autophagosomes (Fig. 1c), and the accumulation of ATG8- and ATG12-positive puncta (Fig. 2d and j). The frequent colocalization of these puncta with ER aggregates indicates that ER stress not only initiates autophagy but also provides the membrane platforms necessary for autophagosome formation (Fig. 2d to f and j to l). Through autophagy, damaged organelles such as chloroplasts are degraded, reducing cellular stress and recycling valuable macromolecules (This study; Wei et al. 2022; Dermastia et al. 2023; Inaba et al. 2023). This degradative response may play a dual role; while it helps the host maintain homeostasis, it also generates metabolic intermediates that the pathogen can exploit.

PPT phytoplasma-induced autophagy was accompanied by significant changes in lipid metabolism of infected tomato plants. Lipidomic analyses revealed extensive degradation of chloroplast-derived lipids (eg, MGDG and PG) and a reduction in photosynthetic membrane components (Fig. 3b and c; Figure S1), consistent with autophagic turnover of damaged chloroplasts induced by PPT phytoplasma infection (Fig. 1a; Wei et al. 2022). Simultaneously, ER-derived phospholipids such as PC and PE increased (Fig. 3c), suggesting enhanced membrane biogenesis and ER remodeling. Moreover, a significant accumulation of TAGs and LDs was observed in infected tissues (Figs. 3a, 4a and b, and 5g to i), reflecting a shift toward energy storage and stress adaptation. Inhibition of autophagy in phytoplasma-infected plants reduced LD mobilization, leading to LD clustering and coalescence (Fig. 4g and h) and significantly lowered phytoplasma titer (Figure S2), indicating that autophagy-driven lipid mobilization is integral to pathogen proliferation. These observations suggest that autophagy is required for LD turnover and that phytoplasmas may exploit this process to access host lipids essential for their survival and proliferation.

Autophagy plays a crucial role in cellular defense by enabling hosts to break down damaged organelles and invading microbes through a process known as xenophagy (Kwon and Song 2018). However, many intracellular pathogens have evolved sophisticated mechanisms to evade, exploit, or reprogram autophagy and ER–associated pathways to promote their replication and persistence. In animal systems, numerous bacteria and viruses exploit host processes to their benefit. For example, *Legionella pneumophila* remodels ER-derived membranes to form *Legionella*-containing vacuoles, where it replicates while being shielded from lysosomal degradation (Horwitz 1983; Escoll et al. 2013). *Coxiella burnetii* establishes a large, autophagosome-like parasitophorous vacuole that supports replication (Romano et al. 2007), while *Brucella abortus* hijacks ER exit sites to create its replicative niche (Celli et al. 2003). *Salmonella enterica* and *Listeria monocytogenes* modulate autophagy recognition systems to evade degradation, either by suppressing ubiquitin tagging or altering surface markers (Birmingham et al. 2007; Mostowy et al. 2011). Viral pathogens also exploit these pathways: *Hepatitis C virus* (HCV) and *Dengue virus* (DENV) co-opt ER membranes to form membranous webs that serve as replication platforms (Miller and Krijnse-Locker 2008; Heaton and Randall 2010), while SARS-CoV-2 manipulates autophagy flux and ER stress responses to enhance its replication and immune evasion (Khan et al. 2024).

In plant systems, viruses have convergently evolved similar strategies. *Turnip mosaic virus* (TuMV) and *Barley stripe mosaic virus* (BSMV) suppress the host's selective autophagy, thereby preventing the degradation of viral proteins and promoting systemic infection (Yang et al. 2018; Li et al. 2020). Unlike animal pathogens that reside in vacuoles or plant viruses that remodel ER membranes, phytoplasmas lack defined replication compartments. Rather than constructing vacuole- or ER-bound niches, phytoplasmas may exploit host ER remodeling and autophagy-related lipid turnover to mobilize lipids and create a nutrient-rich environment within phloem sieve elements, thereby supporting intracellular survival and proliferation.

Another key mechanistic insight from this study is the identification of a MGL-like enzyme (PPT-lipase) encoded in the PPT phytoplasma genome. Bioinformatic analysis revealed that the gene harbors conserved lipase motifs, including the catalytic triad characteristic of the α/β-hydrolase fold (Fig. 7a and c), supporting a putative lipolytic function. PPT-lipase lacks a signal peptide (Table S4), suggesting that the enzyme is retained within the phytoplasma cytoplasm rather than secreted into the host cell. Immunodetection in infected tomato tissues using the anti-PPT-lipase antibody confirmed PPT-lipase expression and revealed its localization near ER-associated structures and neutral lipids, placing it in proximity to host lipid trafficking sites (Figure S3). This spatial pattern suggests a strategic interface for the uptake and internal processing of host-derived lipid intermediates.

Functional assays in heterologous systems demonstrated that PPT-lipase actively modulates lipid homeostasis. In *S. cerevisiae*, expression of WT PPT-lipase reduced BODIPY 493/503 fluorescence compared to the GUS control, and catalytic mutant showed elevated TAG accumulation (Fig. 8b). Similarly, transient expressions in *N. benthamiana* decreased TAG content (Fig. 8c; Table S2). These findings indicate that PPT-lipase possesses intrinsic lipolytic activity that depends on an intact catalytic triad. Although substrate specificity has yet to be determined biochemically, structural similarity to MAG lipase–like enzymes and the observed shifts in lipid profile suggest that PPT-lipase most likely acts on MAG or related neutral lipid intermediates downstream of TAG hydrolysis. Comparable lipid-modifying activity has been reported for a homologous enzyme from Malaysian periwinkle yellows phytoplasma, which exhibited phospholipase A1–like activity in heterologous expression systems (Gedvilaite et al. 2014), supporting conservation of catalytic function among phytoplasma lipases. These findings provide a possible mechanistic link between PPT phytoplasma-induced autophagy/lipid remodeling and PPT-lipase function.

Genome analysis indicates that the PPT phytoplasma lacks canonical bulk TAG lipases capable of initiating large-scale TAG hydrolysis. Therefore, the pathogen is likely dependent on host lipid mobilization pathways, such as cytosolic lipases or autophagy-mediated lipophagy, to generate lipid intermediates. In this model, PPT-lipase would not serve as the primary enzyme driving global TAG breakdown but instead function downstream of host-initiated lipid mobilization by processing host-derived MAG or related intermediates at the phytoplasma–host interface. Consistent with this hypothesis, TAG levels remain elevated in infected tomato tissues (Fig. 3a), whereas neutral lipid signals are enriched in proximity to phytoplasma cells (Figs. 4a and b and 5g to i), suggesting spatially localized lipid remodeling rather than global TAG depletion. PPT-lipase alone is unlikely to explain the broad lipid remodeling observed during infection. Other phytoplasma-encoded factors, including secreted effectors, together with infection-induced host stress pathways, may also regulate host lipases, LD dynamics, and autophagy-associated lipid metabolism. Such coordination between host lipid mobilization and pathogen-associated lipase activity would enable phytoplasmas to access membrane precursors and energy-rich lipid components despite limited de novo lipid synthesis, thereby supporting survival and proliferation within host tissues.

The genomic context of PPT-lipase further supports its potential relevance to phytoplasma adaptation. The PPT phytoplasma genome contig containing the PPT-lipase gene also features prophage-derived remnants, including *fliA* and *himA*, suggesting it may be a genomic island. Such regions are common in phytoplasma genomes and are often associated with horizontal gene transfer and adaptive evolution (Bai et al. 2006; Wei et al. 2008; Šeruga Musić et al. 2019, 2025). Many phytoplasma effectors, including members of the SAP (secreted AY-WB protein) and PHYL families, are located within these mobile genetic elements (Bai et al. 2006; MacLean et al. 2011; Iwabuchi et al. 2020). The placement of PPT-lipase within this prophage-derived region suggests it may have been acquired through horizontal gene transfer and could play a role in phytoplasma adaptation and interactions with hosts.

This study highlights the interconnected roles of the ER, autophagy, and lipid metabolism in shaping the plant–phytoplasma interaction and identifies PPT-lipase as a putative enzyme that facilitates pathogen access to host-derived nutrients. Future work should focus on directly characterizing PPT-lipase substrate specificity, elucidating lipid transport mechanisms, and exploring potential cooperation between phytoplasma and host lipolytic enzymes to fully unravel the metabolic strategies underpinning phytoplasma infection.

## Supplementary Material

kiag628_Supplementary_Data

## Data Availability

All the relevant data are provided in the main text (Figs 1 to 8) and its supporting information (Figures S1 to S4; Tables S1 to S4).

## References

[kiag628-B1] Bai X et al 2006. Living with genome instability: the adaptation of phytoplasmas to diverse environments of their insect and plant hosts. J Bacteriol. 188:3682–3696. 10.1128/JB.188.10.3682-3696.2006. 16672622 PMC1482866

[kiag628-B2] Birmingham CL et al 2007. Listeria monocytogenes evades killing by autophagy during colonization of host cells. Autophagy. 3:442–451. 10.4161/auto.4450. 17568179

[kiag628-B3] Birmingham CL, Smith AC, Bakowski MA, Yoshimori T, Brumell JH. 2006. Autophagy controls Salmonella infection in response to damage to the Salmonella-containing vacuole. J Biol Chem. 281:11374–11383. 10.1074/jbc.M509157200. 16495224

[kiag628-B4] Celli J et al 2003. Brucella evades macrophage killing via VirB-dependent sustained interactions with the endoplasmic reticulum. J Exp Med. 198:545–556. 10.1084/jem.20030088. 12925673 PMC2194179

[kiag628-B5] Cui YL et al 2011. The GDC1 gene encodes a novel ankyrin domain-containing protein that is essential for grana formation in Arabidopsis. Plant Physiol. 155:130–141. 10.1104/pp.110.165589. 21098677 PMC3075748

[kiag628-B6] Deretic V, Saitoh T, Akira S. 2013. Autophagy in infection, inflammation and immunity. Nat Rev Immunol. 13:722–737. 10.1038/nri3532. 24064518 PMC5340150

[kiag628-B7] Dermastia M et al 2023. Candidate pathogenicity factor/effector proteins of ‘*Candidatus* Phytoplasma solani’ modulate plant carbohydrate metabolism, accelerate the ascorbate–glutathione cycle, and induce autophagosomes. Front Plant Sci. 14:1232367. 10.3389/fpls.2023.1232367. 37662165 PMC10471893

[kiag628-B8] Drcelic M, Skiljaica A, Polak B, Bauer N, Seruga Music M. 2024. ‘*Candidatus* Phytoplasma solani’ predicted effector SAP11-like alters morphology of transformed arabidopsis plants and interacts with AtTCP2 and AtTCP4 plant transcription factors. Pathogens. 13:893. 10.3390/pathogens13100893. 39452764 PMC11510232

[kiag628-B9] Escoll P, Rolando M, Buchrieser C. 2016. Modulation of host autophagy during bacterial infection: sabotaging host munitions for pathogen nutrition. Front Immunol. 7:81. 10.3389/fimmu.2016.00081. 26973656 PMC4776121

[kiag628-B10] Escoll P, Rolando M, Gomez-Valero L, Buchrieser C. 2013. From amoeba to macrophages: exploring the molecular mechanisms of Legionella pneumophila infection in both hosts. In: Molecular mechanisms in legionella pathogenesis. Springer, Berlin, Heidelberg. p. 1–34. 10.1007/82_2013_351 23949285

[kiag628-B11] Fan J, Andre C, Xu C. 2011. A chloroplast pathway for the de novo biosynthesis of triacylglycerol in Chlamydomonas reinhardtii. FEBS Lett. 585:1985–1991. 10.1016/j.febslet.2011.05.018. 21575636

[kiag628-B12] Fan J, Yu L, Xu C. 2019. Dual role for autophagy in lipid metabolism in Arabidopsis. Plant Cell. 31:1598–1613. 10.1105/tpc.19.00170. 31036588 PMC6635848

[kiag628-B13] Galluzzi L et al 2017. Molecular definitions of autophagy and related processes. EMBO J. 36:1811–1836. 10.15252/embj.201796697. 28596378 PMC5494474

[kiag628-B14] Gedvilaite A, Jomantiene R, Dabrisius J, Norkiene M, Davis RE. 2014. Functional analysis of a lipolytic protein encoded in phytoplasma phage based genomic island. Microbiol Res. 169:388–394. 10.1016/j.micres.2013.08.007. 24168924

[kiag628-B15] Grininger C et al 2021. Structural changes in the cap of Rv0183/mtbMGL modulate the shape of the binding pocket. Biomolecules. 11:1299. 10.3390/biom11091299. 34572512 PMC8472722

[kiag628-B16] Heaton NS, Randall G. 2010. Dengue virus-induced autophagy regulates lipid metabolism. Cell Host Microbe. 8:422–432. 10.1016/j.chom.2010.10.006. 21075353 PMC3026642

[kiag628-B17] Hogenhout SA et al 2008. Phytoplasmas: bacteria that manipulate plants and insects. Mol Plant Pathol. 9:403–423. 10.1111/j.1364-3703.2008.00472.x. 18705857 PMC6640453

[kiag628-B18] Horwitz MA . 1983. Formation of a novel phagosome by the Legionnaires' disease bacterium (Legionella pneumophila) in human monocytes. J Exp Med. 158:1319–1331. 10.1084/jem.158.4.1319. 6619736 PMC2187375

[kiag628-B19] Hoshi A et al 2009. A unique virulence factor for proliferation and dwarfism in plants identified from a phytopathogenic bacterium. Proc Natl Acad Sci USA. 106:6416–6421. 10.1073/pnas.0813038106. 19329488 PMC2669400

[kiag628-B20] Huang J, Brumell JH. 2014. Bacteria–autophagy interplay: a battle for survival. Nat Rev Microbiol. 12:101–114. 10.1038/nrmicro3160. 24384599 PMC7097477

[kiag628-B21] Huang X, Yao J, Liu L, Luo Y, Yang A. 2022. Atg8–PE protein-based in vitro biochemical approaches to autophagy studies. Autophagy. 18:2020–2035. 10.1080/15548627.2022.2025572. 35072587 PMC9397461

[kiag628-B22] Inaba J, Kim BM, Zhao Y, Jansen AM, Wei W. 2023. The endoplasmic reticulum is a key battleground between phytoplasma aggression and host plant defense. Cells. 12:2110. 10.3390/cells12162110. 37626920 PMC10453741

[kiag628-B23] Inaba JI, Nagy PD. 2018. Tombusvirus RNA replication depends on the TOR pathway in yeast and plants. Virology. 519:207–222. 10.1016/j.virol.2018.04.010. 29734044

[kiag628-B24] Iriondo MN et al 2023. Effect of ATG12–ATG5-ATG16L1 autophagy E3-like complex on the ability of LC3/GABARAP proteins to induce vesicle tethering and fusion. Cell Mol Life Sci. 80:56. 10.1007/s00018-023-04704-z. 36729310 PMC9894987

[kiag628-B25] Iwabuchi N et al 2020. Functional variation in phyllogen, a phyllody-inducing phytoplasma effector family, attributable to a single amino acid polymorphism. Mol Plant Pathol. 21:1322–1336. 10.1111/mpp.12981. 32813310 PMC7488466

[kiag628-B26] Jing H et al 2025. Genome-wide characterization of monoacylglycerol lipase gene family in soybean and functional analysis of GmMAGLs in storage lipid metabolism and drought resistance. BMC Genomics. 26:625. 10.1186/s12864-025-11813-5. 40597643 PMC12211711

[kiag628-B27] Karlsson M, Contreras JA, Hellman U, Tornqvist H, Holm C. 1997. cDNA cloning, tissue distribution, and identification of the catalytic triad of monoglyceride lipase. J Biol Chem. 272:27218–27223. 10.1074/jbc.272.43.27218. 9341166

[kiag628-B28] Khan A, Ling J, Li J. 2024. Is autophagy a friend or foe in SARS-CoV-2 infection? Viruses. 16:1491. 10.3390/v16091491. 39339967 PMC11437447

[kiag628-B29] Knight M, Braverman J, Asfaha K, Gronert K, Stanley S. 2018. Lipid droplet formation in Mycobacterium tuberculosis infected macrophages requires IFN-γ/HIF-1α signaling and supports host defense. PLoS Pathog. 14:e1006874. 10.1371/journal.ppat.1006874. 29370315 PMC5800697

[kiag628-B30] Kwon DH, Song HK. 2018. A structural view of xenophagy, a battle between host and microbes. Mol Cells. 41:27–34. 10.14348/molcells.2018.2274. 29370690 PMC5792709

[kiag628-B31] Li F et al 2020. A plant RNA virus activates selective autophagy in a UPR-dependent manner to promote virus infection. New Phytol. 228:622–639. 10.1111/nph.16716. 32479643

[kiag628-B32] MacLean AM et al 2011. Phytoplasma effector SAP54 induces indeterminate leaf-like flower development in Arabidopsis plants. Plant Physiol. 157:831–841. 10.1104/pp.111.181586. 21849514 PMC3192582

[kiag628-B33] Maejima K et al 2014. Recognition of floral homeotic MADS domain transcription factors by a phytoplasmal effector, phyllogen, induces phyllody. Plant J. 78:541–554. 10.1111/tpj.12495. 24597566 PMC4282529

[kiag628-B34] Martens S, Fracchiolla D. 2020. Activation and targeting of ATG8 protein lipidation. Cell Discov. 6:23. 10.1038/s41421-020-0155-1. 32377373 PMC7198486

[kiag628-B35] Miller S, Krijnse-Locker J. 2008. Modification of intracellular membrane structures for virus replication. Nat Rev Microbiol. 6:363–374. 10.1038/nrmicro1890. 18414501 PMC7096853

[kiag628-B36] Mizushima N, Komatsu M. 2011. Autophagy: renovation of cells and tissues. Cell. 147:728–741. 10.1016/j.cell.2011.10.026. 22078875

[kiag628-B37] Mostowy S et al 2011. P62 and NDP52 proteins target intracytosolic Shigella and Listeria to different autophagy pathways. J Biol Chem. 286:26987–26995. 10.1074/jbc.M111.223610. 21646350 PMC3143657

[kiag628-B38] Munyaneza JE, Crosslin JM, Lee IM. 2007. Phytoplasmas diseases and insect vectors in potatoes of the Pacific Northwest of the United States. Bull Insectology. 60:181–182.

[kiag628-B39] Musetti R et al 2023. The sieve-element endoplasmic reticulum: a focal point of phytoplasma-host plant interaction? Front Microbiol. 14:1030414. 10.3389/fmicb.2023.1030414. 36819061 PMC9932721

[kiag628-B40] Oshima K et al 2004. Reductive evolution suggested from the complete genome sequence of a plant-pathogenic phytoplasma. Nat Genet. 36:27–29. 10.1038/ng1277. 14661021

[kiag628-B41] Park J, Gu Y, Lee Y, Yang Z, Lee Y. 2004. Phosphatidic acid induces leaf cell death in Arabidopsis by activating the Rho-related small G protein GTPase-mediated pathway of reactive oxygen species generation. Plant Physiol. 134:129–136. 10.1104/pp.103.031393. 14730067 PMC316293

[kiag628-B42] Rantala M, Paakkarinen V, Aro EM. 2018. Analysis of thylakoid membrane protein complexes by blue native gel electrophoresis. J Vis Exp. 139:58369. 10.3791/58369. 30320749 PMC6235366

[kiag628-B43] Romano PS, Gutierrez MG, Berón W, Rabinovitch M, Colombo MI. 2007. The autophagic pathway is actively modulated by phase II Coxiella burnetii to efficiently replicate in the host cell. Cell Microbiol. 9:891–909. 10.1111/j.1462-5822.2006.00838.x. 17087732

[kiag628-B44] Schüssele DS et al 2023. Autophagy profiling in single cells with open source CellProfiler-based image analysis. Autophagy. 19:338–351. 10.1080/15548627.2022.2065617. 35435815 PMC9809960

[kiag628-B45] Šeruga Musić M et al 2019. The genome of ‘Candidatus Phytoplasma solani’ strain SA-1 is highly dynamic and prone to adopting foreign sequences. Syst Appl Microbiol. 42:117–127. 10.1016/j.syapm.2018.10.008. 30455068

[kiag628-B46] Šeruga Musić M, Polak B, Drčelić M, Pei SC, Kuo CH. 2025. Sequencing and comparative analyses of ‘Candidatus Phytoplasma solani’ genomes reveal diversity of effectors and potential mobile units. Microb Genom. 11:001401. 10.1099/mgen.0.001401. 40294126 PMC12047186

[kiag628-B47] Steele S, Brunton J, Kawula T. 2015. The role of autophagy in intracellular pathogen nutrient acquisition. Front Cell Infect Microbiol. 5:51. 10.3389/fcimb.2015.00051. 26106587 PMC4460576

[kiag628-B48] Studer G et al 2020. QMEANDisCo—distance constraints applied on model quality estimation. Bioinformatics. 36:1765–1771. 10.1093/bioinformatics/btz828. 31697312 PMC7075525

[kiag628-B49] Sugio A, Kingdom HN, MacLean AM, Grieve VM, Hogenhout SA. 2011. Phytoplasma protein effector SAP11 enhances insect vector reproduction by manipulating plant development and defense hormone biosynthesis. Proc Natl Acad Sci USA. 108:1254–1263. 10.1073/pnas.1105664108. PMC3228479 22065743

[kiag628-B50] Tan Y et al 2021. Integration of metabolomics and existing omics data reveals new insights into phytoplasma-induced metabolic reprogramming in host plants. PLoS One. 16:e0246203. 10.1371/journal.pone.0246203. 33539421 PMC7861385

[kiag628-B51] Wei W et al 2004. In planta dynamic analysis of onion yellows phytoplasma using localized inoculation by insect transmission. Phytopathology. 94:244–250. 10.1094/PHYTO.2004.94.3.244. 18943972

[kiag628-B52] Wei W et al 2024. iPhyDSDB: phytoplasma disease and symptom database. Biology (Basel). 13:657. 10.3390/biology13090657. 39336085 PMC11429218

[kiag628-B53] Wei W, Davis RE, Jomantiene R, Zhao Y. 2008. Ancient, recurrent phage attacks and recombination shaped dynamic sequence-variable mosaics at the root of phytoplasma genome evolution. Proc Natl Acad Sci USA. 105:11827–11832. 10.1073/pnas.0805237105. 18701718 PMC2515622

[kiag628-B54] Wei W, Davis RE, Nuss DL, Zhao Y. 2013. Phytoplasmal infection derails genetically preprogrammed meristem fate and alters plant architecture. Proc Natl Acad Sci USA. 110:19149–19154. 10.1073/pnas.1318489110. 24191032 PMC3839765

[kiag628-B55] Wei W, Inaba J, Zhao Y, Mowery JD, Hammond R. 2022. Phytoplasma infection blocks starch breakdown and triggers chloroplast degradation, leading to premature leaf senescence, sucrose reallocation, and spatiotemporal redistribution of phytohormones. Int J Mol Sci. 23:1810. 10.3390/ijms23031810. 35163732 PMC8836287

[kiag628-B56] Wu W et al 2012. Salicylic acid-mediated elicitation of tomato defence against infection by potato purple top phytoplasma. Ann Appl Biol. 161:36–45. 10.1111/j.1744-7348.2012.00550.x.

[kiag628-B57] Xu C, Fan J. 2022. Links between autophagy and lipid droplet dynamics. J Exp Bot. 73:2848–2862. 10.1093/jxb/erac003. 35560198

[kiag628-B60] Yang Y, Benning C. 2018. Functions of triacylglycerols during plant development and stress. Curr Opinion Biotechnol. 49:191–198. 10.1016/j.copbio.2017.09.003 28987914

[kiag628-B58] Yang M et al 2018. Barley stripe mosaic virus γb protein subverts autophagy to promote viral infection by disrupting the ATG7–ATG8 interaction. Plant Cell. 30:1582–1595. 10.1105/tpc.18.00122. 29848767 PMC6096602

[kiag628-B59] Yoshitake Y, ohta H, Shimojima M. 2019. Autophagy-mediated regulation of lipid metabolism and its impact on growth in algae and seed plants. Front Plant Sci.. 10:709. 10.3389/fpls.2019.00709. 31214225 PMC6558177

