# Supplementary material for: Potato purple top phytoplasma infection induces autophagy-associated lipid dynamics that support pathogen proliferation
Source: Plant Physiol. 2026 Aug 25;202(1):kiag628. doi: 10.1093/plphys/kiag628 (PMC13535232; doi:10.1093/plphys/kiag628)
Supplement: Supplementary file 1 [file kiag628_supplementary_data.zip › Figs-S1-S4.pdf]

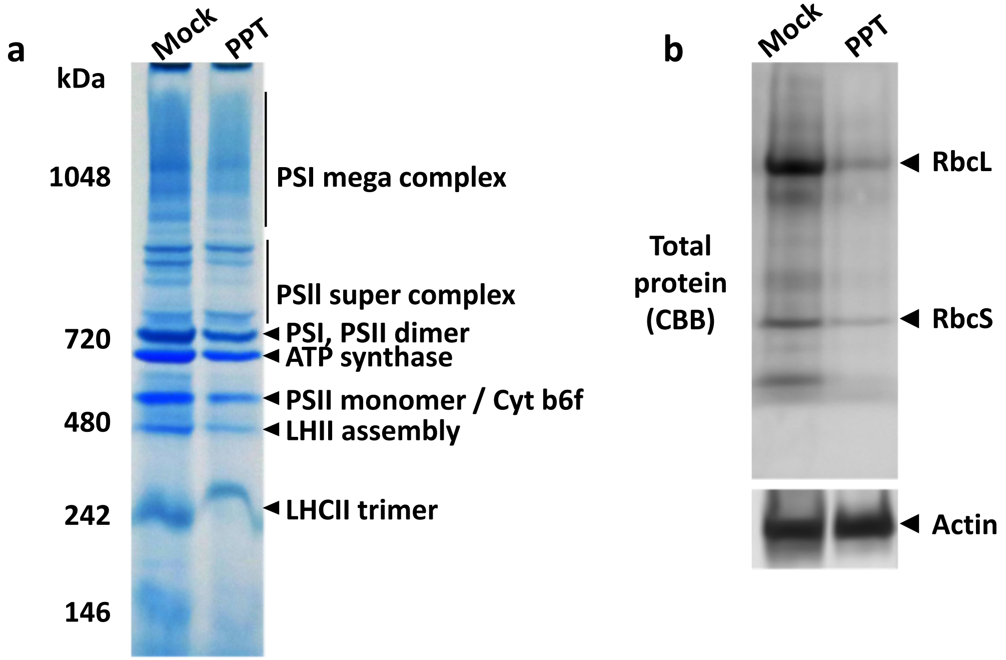

Fig. S1. Chloroplast thylakoid membrane organization is altered in tomato plants infected with potato purple top (PPT) phytoplasma. (a) Blue Native-PAGE (BN-PAGE) analysis of chloroplast thylakoid membrane complexes. Chloroplast fractions were isolated from leaves of mock-treated and PPT phytoplasma-infected plants. Protein complexes were separated by BN-PAGE to assess the integrity of high-molecular-weight thylakoid assemblies. In infected samples, the abundance of several major thylakoid complexes was markedly reduced or appeared destabilized compared with the mock control, indicating disruption of chloroplast membrane organization. (b) SDS-PAGE analysis of Rubisco content. Total leaf proteins from mock and PPT phytoplasma-infected plants were subjected to SDS-PAGE to evaluate Rubisco abundance. The Rubisco large subunit band intensity was substantially lower in infected plants, suggesting impaired photosynthetic capacity consistent with chloroplast structural disruption. Actin immunoblotting confirmed equal protein loading.

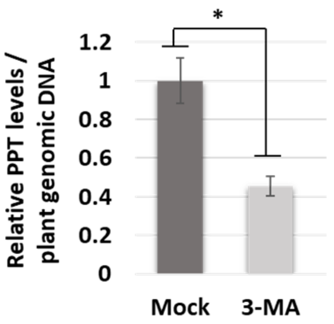

Fig. S2. Inhibition of autophagy reduces potato purple top (PPT) phytoplasma accumulation in infected tomato plants. Quantitative PCR analysis showed that phytoplasma titers were significantly lower in 3-methyladenine (3-MA)–treated infected plants than in untreated infected controls, indicating that autophagy supports phytoplasma proliferation. Data represent mean ± SD from three biological replicates. Statistical significance (*) was determined using Student's t-test ($P < 0.05$).

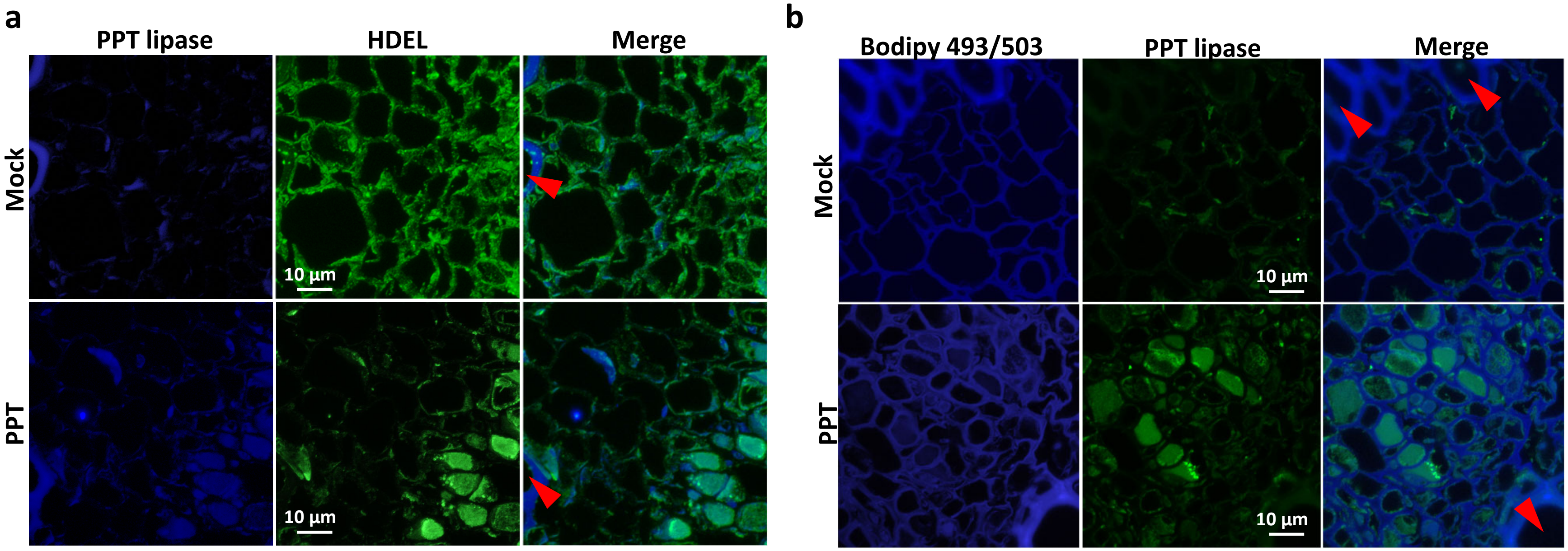

Fig. S3. Subcellular localization of PPT-lipase and its spatial association with host ER and lipid droplets. (a) Confocal micrographs of tomato stem cross-sections immunolabeled with anti–PPT-lipase (blue) and anti-HDEL (ER marker; green). (b) Immunostaining of anti–PPT-lipase (green) alongside BODIPY 493/503 staining of neutral lipid droplets (blue). Upper panels in (a) and (b) represent mock-inoculated controls; lower panels show PPT phytoplasma–infected samples. In infected tissues, PPT-lipase signals are enriched in the phloem and partially colocalize with both the ER network (a, lower right) and neutral lipid droplets (b, lower right). These spatial correlations suggest that PPT-lipase targets ER-associated membranes and host lipid reservoirs during infection. Scale bar = 10 µm.

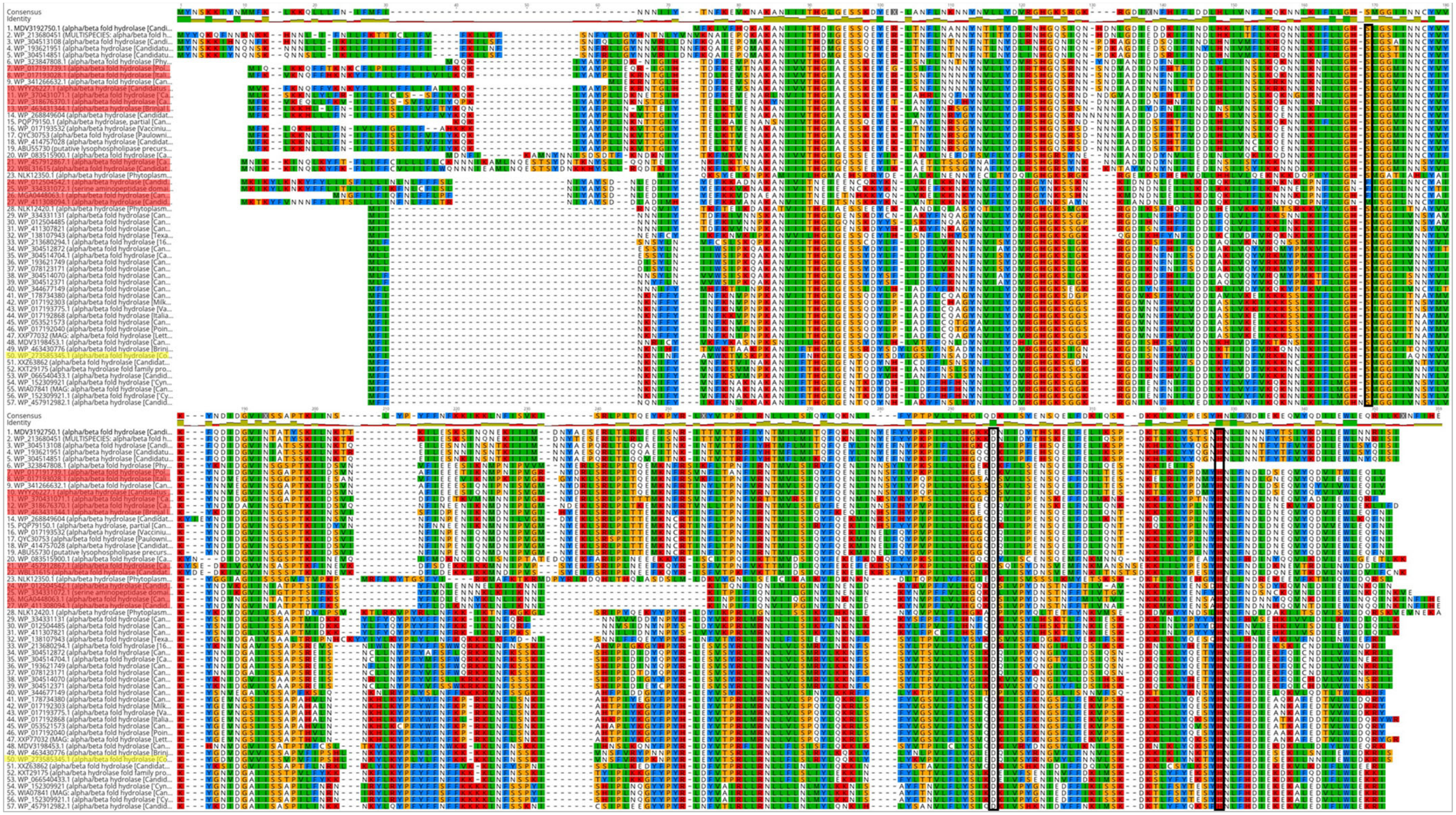

Fig. S4. Multiple sequence alignment of phytoplasma lipases across 57 phytoplasmas. Protein sequences of predicted phytoplasma lipases were aligned using Geneious Primer (2026. 0.2) to assess conservation and domain structure. Conserved residues and motifs are shown across sequences. Potential secreted lipases, identified by predicted signal peptides, are highlighted in red. The conserved catalytic triad (Ser99–Asp208–His242) is marked by black boxes. The entry highlighted in yellow is the lipase identified in this study.
